# 3.3–4.3 GHz efficient continuous class-F gallium nitride power amplifier based on simplified real frequency technique and harmonic tuning

**DOI:** 10.1371/journal.pone.0306738

**Published:** 2024-08-14

**Authors:** Md. Golam Sadeque, Zubaida Yusoff, Mardeni Roslee, Shaiful Jahari Hashim, Azah Syafiah Mohd Marzuki, Jonathan Lees, Farid Zubir, Samir Salem Al-Bawri

**Affiliations:** 1 Department of Electrical and Electronic Engineering, Pabna University of Science & Technology, Pabna, Bangladesh; 2 Faculty of Engineering, Multimedia University, Cyberjaya, Selangor, Malaysia; 3 Faculty of Engineering, Universiti Putra Malaysia, Serdang, Selangor, Malaysia; 4 TM R&D Sdn Bhd, Cyberjaya, Selangor, Malaysia; 5 Centre for High Frequency Engineering, Cardiff University, Cardiff, Wales, United Kingdom; 6 Wireless Communication Centre, Faculty of Electrical Engineering, Universiti Teknologi Malaysia, Johor Bahru, Johor, Malaysia; 7 Space Science Centre, Climate Change Institute, Universiti Kebangsaan Malaysia (UKM), Bangi, Malaysia; Model Institute of Engineering and Technology, INDIA

## Abstract

In order to implement the fifth generation (5G) communication system for a large number of users, the governments of many countries nominated the low 5G frequency band between 3.3 and 4.3 GHz. This paper proposes a wideband RFPA by designing the input matching network (MN) and output MN of the device using the simplified real frequency technique (SRFT) and the harmonic tuning network. The load-pull and source-pull is applied at multiple points for 100 MHz intervals over the bandwidth to obtain the optimum impedances at the output and input of the 10W Gallium Nitride (GaN) Cree CGH40010F device. To verify the design, the RFPA is simulated, and the performance is measured between 3.3 and 4.3 GHz. According to experimental findings, the measured drain efficiency (DE) throughout the whole bandwidth ranged from 57.5 to 67.5% at the output power of 40 dBm. Moreover, at the 1 dB compression point between 39.2 and 42.2 dBm output power, the drain efficiency (DE) achieves a high value of 81.2% with an output power of 42.2 dBm at a frequency of 3.3 GHz. The RFPA can obtain a maximum gain of 12.4 dB at 3.5 GHz. The linearity of the RFPA with a two-tone signal is measured and the value is less than -22 dBc all over the band.

## Introduction

Different generation cellular communication systems have developed since the first generation (1G) system. The latest generation communication system is the fifth generation (5G) communication system. In modern communication systems, the most power-consuming unit is the RF power amplifier (RFPA). So, high efficiency is one of the main requirements of the RFPA. 5G wireless system will offer some new capabilities (high data speed and low latency) and ubiquitous connectivity. So, to provide this service to a huge number of users, the authority will not only convert the existing base station for the 5G system but also use small cell technology as well. The 5G communication system will expand the spectrum of frequency that will have been utilized for mobile communications in order to fulfill the bandwidth needed for transmitting high data rates and enhancing the capacity of data traffic. New spectrums of the 5G system are allocated below 6 GHz as well as above 6 GHz [1]. For IMT systems, almost all nations will use a spectrum below 6 GHz. For a low 5G frequency band, which is below 6 GHz, the government bodies from different countries allow different frequency bands, as shown in Table 1 of [2]. From the table, 4.3 GHz is the maximum frequency, while the lowest is 3.3 GHz for the low 5G frequency band.

**Table 1:** Source-pull and load-pull data.

Frequency (GHz)	*Z*_*L*1_	*I*_*L*1_	*Z*_*S*1_	*I*_*S*1_	Gain	Pout	PAE
			Conj.	Conj.	(dB)	(dBm)	
3.3	29.89+j24	-0.15+0.34i	33.3-j18.9	-0.14–0.25i	10.2	40.2	59.1
3.4	26.8+j15.2	-0.25+0.25i	41.9-j24.1	-0.02–0.27i	10.8	40.8	66.1
3.5	22.6+j7.9	-0.36+0.15i	54.5–28.3i	0.10–0.24i	11.04	41.04	66.0
3.6	23.2+j2.0	-0.37+0.03i	76.0-j26.0	0.26–0.16i	10.9	40.9	63.9
3.7	22.6-j7.9	-0.36–0.15i	100.7-j8.6	0.34–0.04i	11.3	41.3	60.9
3.8	28.4-j9.2	-0.25–0.15i	106.1+37.5i	0.39+0.15i	10.8	40.8	57.5
3.9	36.3-j3.7	-0.15–0.04i	72.0+j67.0	0.37+0.35i	10.3	40.3	57.6
4.0	23-j2.0	-0.37–0.04i	41.4+j67.8	0.29+0.52i	9.8	39.8	64.0
4.1	11.9-j13	-0.54–0.32i	24.0+j63.0	0.22+0.66i	10.0	40.0	71.4
4.2	9.5-j21.9	-0.48–0.54i	16.0+j53.0	0.08+0.74i	9.8	39.8	67.9
4.3	10.1-j28.6	-0.35–0.64i	11.7+j45.5	-0.05+0.77i	9.9	39.9	67.7

As high efficiency is a key requirement of the power amplifier for the wide bandwidth, to improve the efficiency, several modes of RFPA have been investigated till now. Harmonics tuning power amplifier is a good choice for high efficiency. Widely used high efficiency RFPA are class-D, class-E, class-F, inverse class-F and Doherty configuration [3]. To obtain high efficiency, the harmonic impedances should be controlled very precisely. But, it is impossible to control harmonics for the wide bandwidth. Therefore, those amplifiers are suitable for the narrowband RFPA only. To extend the bandwidth, the continuous mode RFPA is introduced in [4]. The wide mode RFPAs are Class-J [5], continuous class-F (CCF) and continuous inverse class-F (CCF-1).

So far, a variety of impedance matching methods have been developed and applied for the matching of the active device. In 1979, the mathematical algorithm recognized as the real frequency technique (RFT) is presented by Carlin and Komiak [6]. In this method, only real frequency impedance data is required and the approximated circuit of the active device is not needed. This algorithm is further developed to simplified real frequency technique (SRFT) in [7] and [8]. Among many impedance matching techniques, SRFT is one of the most accepted methods for the wideband RFPA. In this method, an optimization algorithm is used for impedance matching of the wide bandwidth. The mathematical algorithm is applied for matching network’s (MN) synthesis with L-C ladder passive components. In this study, the CCF RFPA is implemented using the SRFT for the low 5G frequency band with 1 GHz bandwidth. Previously, a conference paper with the same objective presented the simulation results as referenced in [2]. This paper represents an extended version of that earlier work from [2], incorporating the addition of a harmonic tuning network. The design is then simulated and updated with an optimized power amplifier. Finally, the designed and fabricated power amplifier is presented along with the analysis of both simulation and measurement results of the improvised design.

The structure of this paper is prepared in this order. In section 2, the optimum impedance of the active device for the CCF mode RFPA is deduced at the current generator (I-Gen) plane. After the optimum impedance is deduced, the matching network for the impedance at fundamental is designed and it is followed by the design of the matching network at harmonic frequencies. The theoretical values of the MN’s passive components are obtained by applying the SRFT and synthesizing them using the LC ladder circuit. In section 3, the design of the CCF class-F RFPA is built using the proposed methodology in section 2. In section 4, the RFPA simulation is compared to the measurement results. The implemented RFPA achieves drain efficiency (DE) between 57.5% to 67.5%. Section 5 draws the conclusions.

## Materials and methods

### The continuous class-F (CCF)

The harmonic impedances at even and odd frequencies are suitably terminated at the I-Gen plane to provide the conventional class-F mode RFPA. As a result, for the conventional class-F, the drain current i_D_ is a half-rectified sinusoidal waveform expressed by Eq (1) and the drain voltage v_D_ is a square waveform up to the third harmonic represented by Eq (2). The standard class-F mode RFPA provides excellent efficiency for the narrow band by properly terminating the harmonic impedances, but it is not suitable for the operation of the wideband. The continuous class-F (CCF) RFPA is a good alternative for the wide bandwidth and high efficiency requirements of RFPA. This alternative choice provides multiple impedance solutions for maintaining the same efficiency which is achieved by multiplying the drain voltage (up to third harmonics) of class-F RFPA with a factor (1-γsinθ) as obtained in Eq (3).


$$\begin{array}{l} i_{D}=I_{\max}\cos\theta ,-\frac{\pi }{2}<\theta <\frac{\pi }{2}\\ \;=0,-\pi <\theta \leq -\frac{\pi }{2};\;\frac{\pi }{2}\leq \theta \leq \pi \end{array}$$ (1)

$$v_{D}=1-\frac{2}{\sqrt{3}}\cos\theta +\frac{1}{3\sqrt{3}}\cos3\theta$$ (2)

$$v_{D}=(1-\frac{2}{\sqrt{3}}\cos\theta +\frac{1}{3\sqrt{3}}\cos3\theta )(1-\gamma \sin\theta )$$ (3)

Depending on the value of γ, the load impedances represented by the CCF up to third harmonics are derived from the equations of the drain voltage and the drain current [9] and the results are as follows:

$$Z_{F}=R_{opt}\frac{2}{\sqrt{3}}+j\gamma R_{opt}$$

$$Z_{2,F}=-jR_{opt}\frac{7\sqrt{3}\pi }{24}\gamma$$

$$Z_{3,F}=\infty$$

$$R_{opt}=\frac{V_{DC}-V_{knee}}{I_{max}/2}$$ (4)

where *Z*_*F*_, *Z*_2*F*_, and *Z*_3*F*_ are the impedance at the I-Gen plane for the fundamental frequency, the second harmonic frequency and the third harmonic frequency, respectively. R_opt_ is the ideal impedance for the typical class-B operation, *V*_*knee*_ is the knee voltage, which separates the transistor’s linear and saturation regions and *I*_*max*_ is the transistor’s maximum drain current. Due to the parasitic effect and packaging components, the I-Gen plane optimum impedance must include the parasitic capacitances and therefore a new reference plane is established which is called the package plane. This new package plane is used for the design of the output MN.

### Simplified real frequency technique (SRFT)

The SRFT method was originally created by Yaman and Carlin [7]. This method was mostly focused on the synthesis of the lump components, the LC network. Then, for the synthesis of matching network with the distributed networks involving commensurate transmission lines, the SRFT method was improved and enhanced. The MN’s input reflection coefficient, *S*_11_(*p*), for the SRFT method may be expressed as follows [7, 10–12]:

$$S_{11}=\frac{h(p)}{g(p)}=\frac{h_{0}+h_{1}p+\cdots +h_{n}p^{n}}{g_{0}+g_{1}p+\cdots +g_{n}p^{n}}$$ (5)

where *h(p)* is the polynomials of order *n* at the numerator and *g(p)* is the polynomials of order *n* at the denominator. The number of reactive elements employed in the matching network is determined by the order of the polynomial. The *g(p)* is called the Hurwitz polynomial, which is calculated using the MATLAB software from the polynomial *h(p)*. It is necessary to choose a good initial value of *h(p)* to run the program efficiently. The initial value of *h(p*) is chosen to be +1 or -1. Using the Belevitch representation, the other scattering parameters of the MN (sometimes referred to as the equalizer) may be represented as:

$${S_{12}=S}_{21}=\pm \frac{f(p)}{g(s)}$$

$$S_{22}=-{\left(-1\right)}^{k}\frac{h(-p)}{g(p)}$$ (6)

It should be noted that k is an integer that determines the order of the transmission zeros at the origin. For low pass LC ladder MN, *f*(p) = 1. To assess how well the active device and the 50 Ω load are matched, the transducer power gain (TPG) is determined. The TPG may be stated as follows [8]:

$$TPG(\omega )=\frac{{\left|S_{21}\right|}^{2}(1-{\left|\Gamma_{L}\right|}^{2})}{{\left|1-S_{11}\Gamma_{L}\right|}^{2}}$$ (7)

where Γ_*L*_ is the load side’s normalized reflection coefficient. The equalizer network must be lossless. So, for this condition, |*S*_21_|^2^ can be expressed in writing as follows:

$${\left|S_{21}\right|}^{2}=1-{\left|S_{11}\right|}^{2}=\frac{{\left|f(p)\right|}^{2}}{g(p)g(-p)}$$ (8)

where g(p)g(−p)=h(p)h(−p)+f(p)f(−p). As a result, the TPG is determined by inserting the value of |*S*_21_|^2^ in Eq (7). So, the TPG becomes

$$TPG(\omega )=\frac{\left[f(p)f(-p)](1-{\left|\Gamma_{L}\right|}^{2}\right)}{h(p).h(-p).[{1+|\Gamma_{L}|}^{2}]+f(p)f(-p)-2real[\Gamma_{L}.h(p)g(-p)]}$$ (9)

Then, the least square approach is used to make the TPG as flat as feasible over the band of interest through MATLAB programming. After that, the error, *(δ)* equation can be estimated using the formula below:

$$\delta =\sum_{i=1}^{m}T(\omega_{i})-T_{0}$$ (10)

where $T_{\left(\omega_{i}\right)}$, *T*_0_ and *m* stands for TPG at the frequency *ω*_*i*_, the desired flat gain level and the sampling frequencies over the passband, respectively. The error *(δ)* is calculated can be minimized by optimizing the polynomial’s coefficient of *h(p)*. The Levenberg-Marquard method is used to determine the increment of coefficient *Δh*. Then, the optimized TPG is then calculated utilizing Eq (9). As the optimized coefficient of *S*_11_ is obtained from the polynomial *h(p)* and *g(p)*, then the MN’s input impedance is calculated using the Eq (11).


$$Z(p)=\frac{1+S_{11}(p)}{1-S_{11}(p)}$$ (11)

Once the input impedance is obtained, the next section explains how the matching network (MN) is synthesized using a low-pass LC ladder network with a resistive termination. The normalized components value is determined by applying MATLAB programming.

### Matching network (MN) synthesis

**Fig 1:**
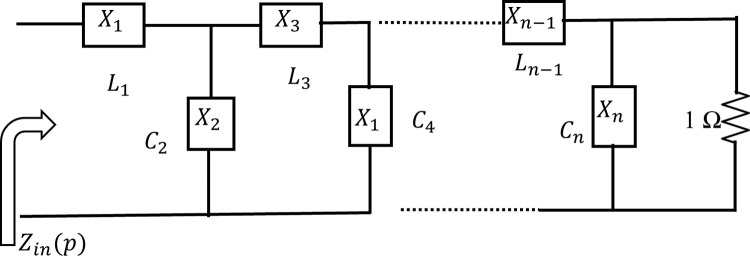
n^th^ order low pass structure.

**Fig 2:**
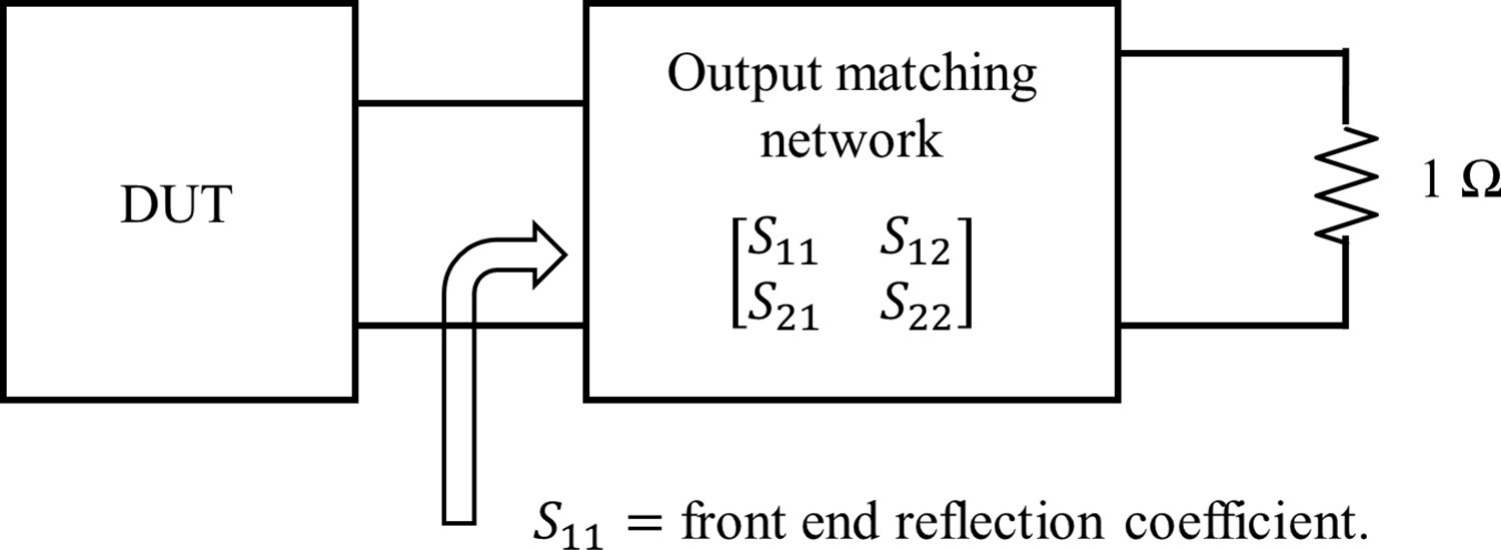
Normalized MN from the active device to reference impedance.

Let us consider a simple n^th^-order low-pass LC ladder network is terminated with a unit resistor, as shown in Fig 1, where *X*_*n*_ denotes either a series inductor or a shunt capacitor. Then, the input impedance *Zi*_*n*(*p*)_ of the low pass LC ladder network is represented by two polynomials [10] as in Eq (12);

$$Z_{in(p)}=\frac{N(p)}{D(p)}$$ (12)

where *N*(*p*) is the numerator and *D*(*p*) is the normalized input impedance. From Fig 2, the low-pass LC ladder network is presented as an equivalent two-port network. The two-port network can be characterized using the normalized scattering parameter.

A considerable simplification in characterizing the lossless two-port network is achieved by Belevitch, who showed that the scattering coefficients of the two-port network can be denoted using only two polynomials *h*(*p*) and *g*(*p*) and a unimodular constant *f*(*p*) = *p*^*k*^ (for low pass case *k* =0). So, the relation between the input scattering parameter of the low-pass ladder network and the input impedance of the network is as follows:

$$S_{11}(p)=\frac{Z_{in}(p)-1}{Z_{in}(p)+1}=\frac{N(p)-D(p)}{N(p)+D(p)}=\frac{h(p)}{g(p)}$$

$${S_{12}=S}_{21}=\frac{{f(p)=p}^{k}}{g(p)}=\frac{1}{g(p)}$$

$$S_{22}=-\frac{h(-p)}{g(p)}$$ (13)

Generally, the RFPA is designed for a reference of 50 Ω impedance at the input and output port. Therefore, the output MN has to be designed between the optimum load impedance of the transistor device-under-test (DUT) and at a reference of 50 Ω impedance, as shown in Fig 2, and the input MN has to be designed between a reference 50 Ω impedance and the optimum source impedance of the device as described in details in section 3. This type of matching problem is called a single matching problem since it is designed between the resistive and complex impedance.

The optimized input reflection coefficient, *S*_11_, of the MN is obtained by applying the SRFT algorithm. The broadband MN is designed considering the input impedance calculated by the SRFT technique. After obtaining the *S*_11_, the input impedance of the MN is calculated by using Eq (11). For the low pass ladder technique, the input impedance can be expressed as below:

$$Z_{in}(p)=Z_{1}(p)+\frac{1}{Y_{2}(p)+\frac{1}{Z_{3}(p)+\frac{1}{Y_{4}(p)+\cdots }}}$$ (14)

In this technique, the MN’s normalized component value is calculated by the long division and it is done by applying the MATLAB programming. Then, the normalized component value is transformed into the denormalized values by applying Eq (15) using a proper scaling factor and the operating frequency of the RFPA.

$$C_{k}={C_{k}}^{\prime }\left(\frac{{\omega_{m}}^{\prime }}{\omega_{m}}\right)\frac{R^{\prime }}{R}$$

$$L_{k}={L_{k}}^{\prime }\left(\frac{{\omega_{m}}^{\prime }}{\omega_{m}}\right)\frac{R}{R^{\prime }}$$ (15)

where *C*_*k*_′, *L*_*k*_′ are the capacitor and inductor values for the normalized design and *C*_*k*_ and *L*_*k*_ are the inductor and capacitor values for the scaled design. *R* is the desired resistance level of one termination while *R*′ is the corresponding resistance of the normalized design. The value of *ω*_*m*_′ = 1 is used for the corresponding frequency of the normalized design.

### Design implementation

**Fig 3:**
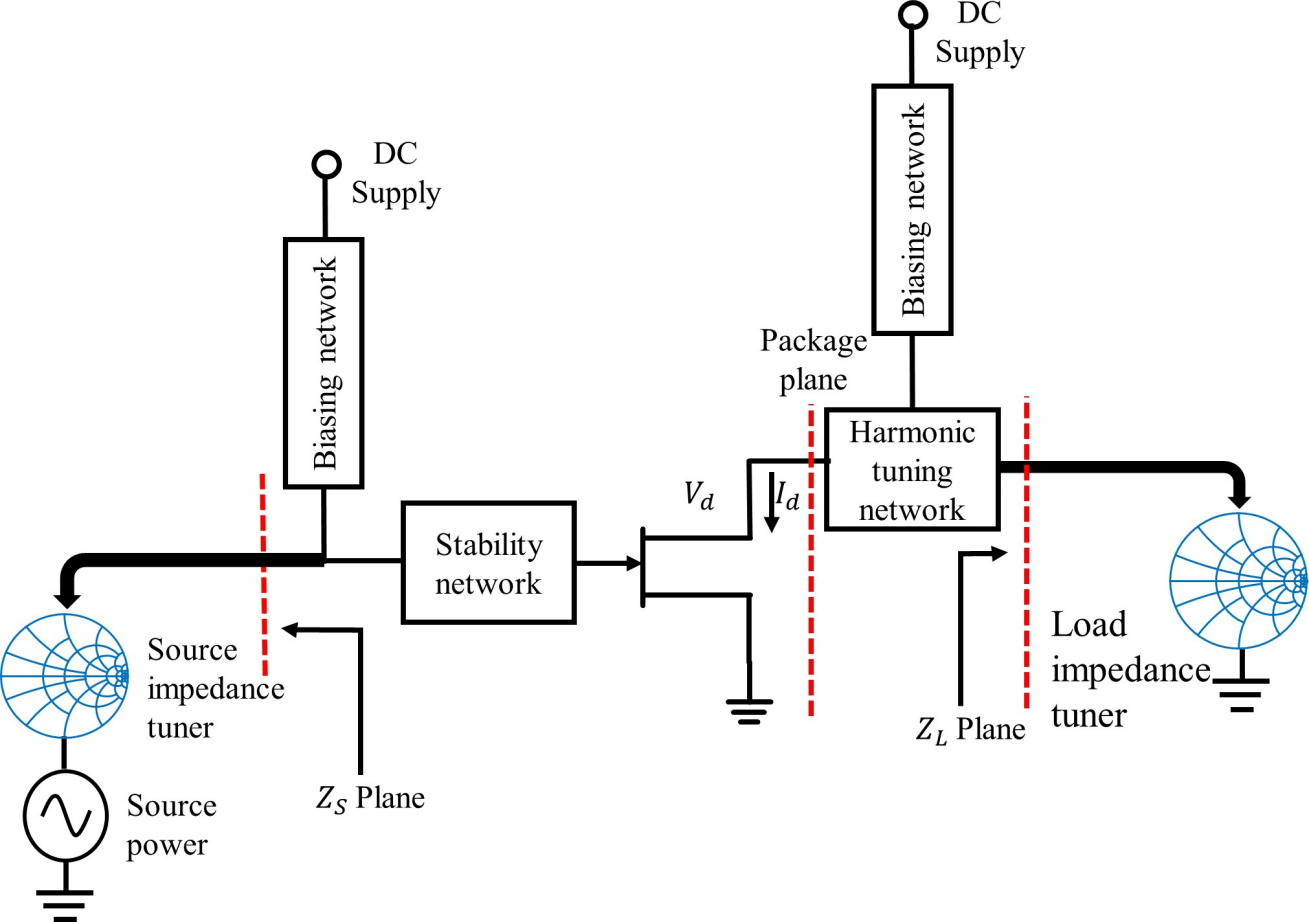
Load-pull and source-pull setup.

The design methodology of the RFPA has been described in detail in section 2. Using that methodology, the RFPA is built with a 10 W Gallium Nitride (GaN) Cree CGH40010F device. Referring to the datasheet, the device can be biased at 28 V DC and the 60 mA biasing current is chosen for operating the RFPA in the CCF mode. The first step of implementing the RFPA is to choose the biasing network. Several types of biasing networks can be used depending on the applications [13]. In this design, the radial stub and quarter-wave microstrip line are used to build the biasing network as in [13]. Next, the Cree device must be checked to achieve unconditionally stable over the required frequency band. The CGH40010F device is initially unstable between 3.3 GHz to 4.3 GHz. So, to stabilize the Cree CGH40010F device in this frequency band, the parallel combination of the RC network is used at the gate of the device. When unconditional stability is achieved then the next step is the determination of optimum impedance at the input and output of the Cree device. To find the optimum load and source impedance of the Cree device, the load-pull and source-pull technique is applied. Before performing this technique, the harmonic tuning network (HTN) is designed to control the harmonics impedance at the output of the device using the impedance buffer concept as described in detail in [9]. Then, the load-pull and source-pull circuit is used to find the optimum impedance at *Z*_*S*_ and *Z*_*L*_ plane, as shown in Fig 3.

In this study, the RFPA is designed for a wide bandwidth and the MN of this RFPA is designed using SRFT. For this matching technique, optimum impedances at multiple points within the frequency band are needed. So, the load-pull and source-pull technique is applied at multiple points of the desired frequency band whereas most of the impedance matching techniques mentioned in the literature used only one optimum impedance at the middle frequency of the band. The load-pull and source-pull data at *Z*_*S*_ and *Z*_*L*_ plane is listed in Table 1. Using the 50 Ω reference impedance, the optimal impedance is changed into the reflection coefficient. Total of eleven points are chosen for optimizing the MN for the whole frequency band, as shown in Table 1.

After obtaining the optimum impedances, the SRFT algorithm mentioned in the methodology section is applied to find the optimized *S*_11_ all over the band. The SRFT algorithm starts by initializing the numerator polynomial h(p) for the reflection of Eq (5). Afterwards, the polynomial g(p), which is a Hurwitz polynomial, is computed using the polynomial h(p). After that, the TPG is calculated using Eq (9). The process is iterated until a flat TPG across the frequency range is achieved. When the response is satisfactory, the input impedance is calculated by applying Eq (11).

**Fig 4:**
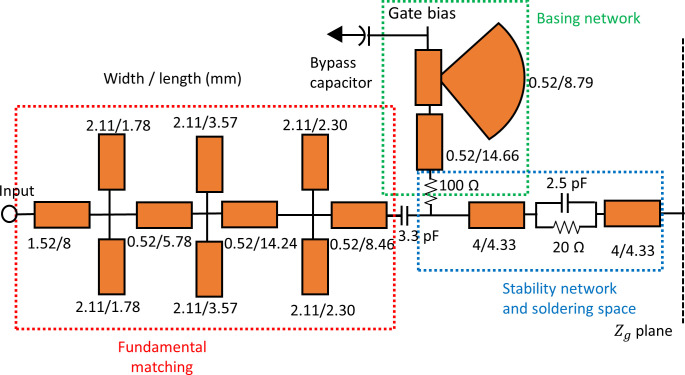
The optimized output MN.

In the next step, the low pass ladder technique mentioned in the methodology section is applied to synthesize the output MN and the normalized component values are obtained. The normalized lump components are denormalized using Eq (15). Table 2 presents the listed values of the lump components, including their normalized, denormalized, and optimized values. Finally, the lump components are converted to microstrip lines using Richards’ transformation [14]. Then, the final dimensions of the microstrip lines are optimized using Keysight Advanced Design System (ADS). The optimized output MN is shown in Fig 4, which consists of HTN (indicated by a red box) and the fundamental MN (indicated by the blue box).

**Table 2:** Normalized, denormalized, and optimized components’ values of the output MN.

Component type	Normalized component values	Denormalized values	Optimized values
L1	0.2391	0.44 nH	0.63 nH
C2	3.2313	2.39 pF	1.88 pF
L3	0.8408	1.56 nH	1.83 nH
C4	2.4409	1.81 pF	1.38 pF

**Fig 5:**
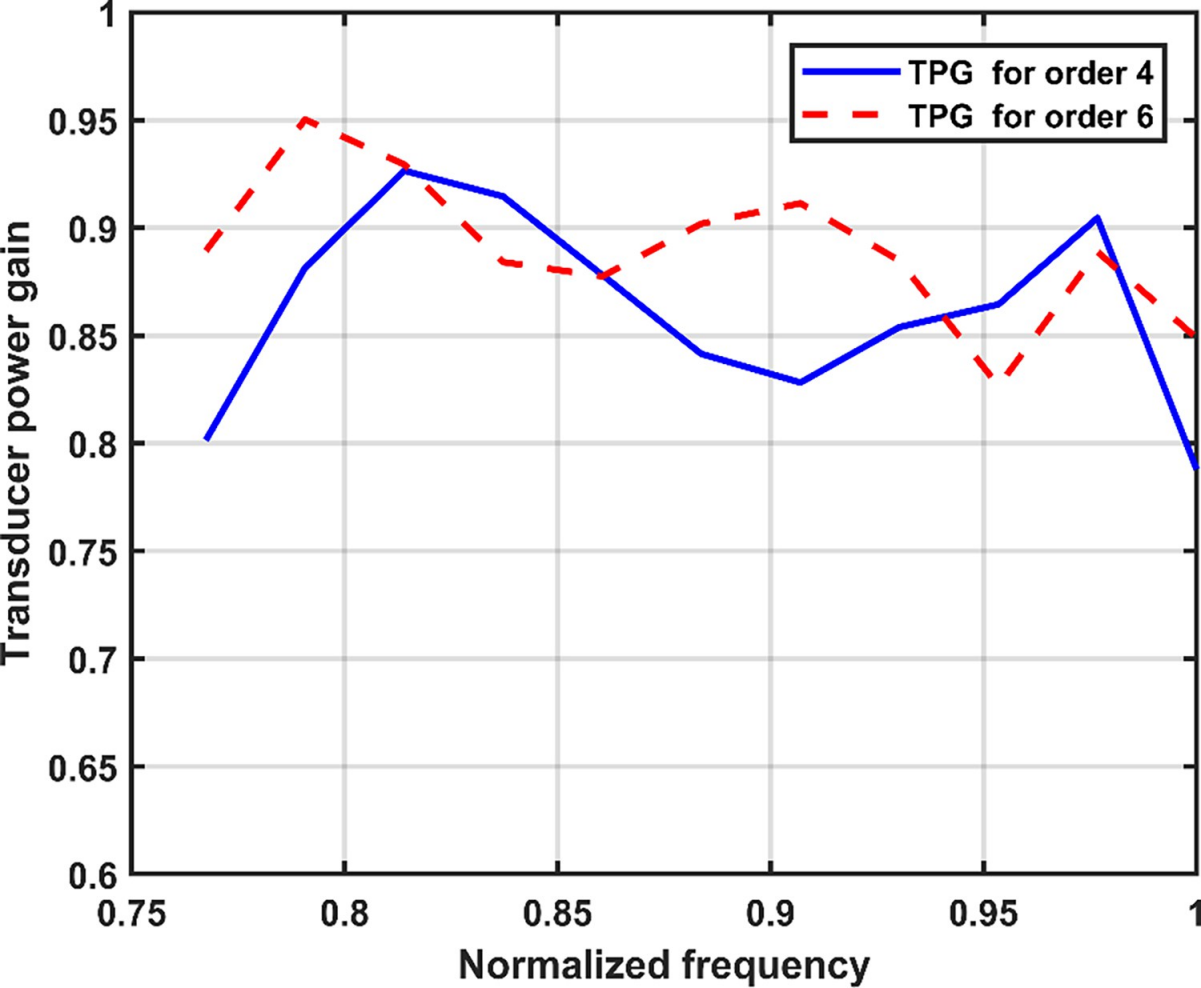
TPG versus normalized frequency.

The input MN is also very critical for the wideband RFPA [15], and it can affect the performance of the RFPA. The input MN consists of the stability network and fundament frequency MN. The parallel combination RC is chosen as a stability network for the device. In the design of the input MN, the role of harmonics impedance in enhancing efficiency is comparatively less significant when compared to the fundamental impedance [3]. Therefore, the design of the input matching network focuses on selecting the optimal fundamental impedance. The optimal source impedance, as listed in Table 1, is utilized for the input matching network design. Additionally, the SRFT algorithm employed for the design of the output matching network is also applied to design the input matching network. In this scenario, a fourth-order low-pass LC ladder MN is selected for the input, and the corresponding initialization of the h(p) polynomial is performed. However, the performance in terms of TPG is unsatisfactory across the frequency band, as depicted in Fig 5. Therefore, to get better performance, the order of the low pass MN was increased to six. Then, the same process is carried out again to calculate TPG as for the output MN, and satisfactory performance was attained throughout the bandwidth, as shown in Fig 5. Subsequently, the input reflection coefficient *S*_*11(*_*p)* is obtained by utilizing the polynomials *h(p)* and *g(p)* in accordance with Eq (5). Following that, the input impedance is determined from the reflection coefficient using Eq (11). Afterward, the normalized matching network (MN) is obtained from the input impedance using low pass LC ladder synthesis, which is achieved through MATLAB programming. The normalized and denormalized values of the lump components are presented in Table 3.

**Table 3:** Values of the components in the input matching network (MN), including their normalized, denormalized, and optimized values.

Component type	Normalized component value	Denormalized value	Optimized value
L1	3.9427	7.30 nH	5.69 nH
C2	0.6036	0.45 pF	0.56 pF
L3	5.3267	9.86 nH	9.07 nH
C4	1.3241	0.98 pF	0.80 pF
L5	1.6244	3.01 nH	3.4 nH
C6	1.3814	1.02 pF	0.84 pF

**Fig 6:**
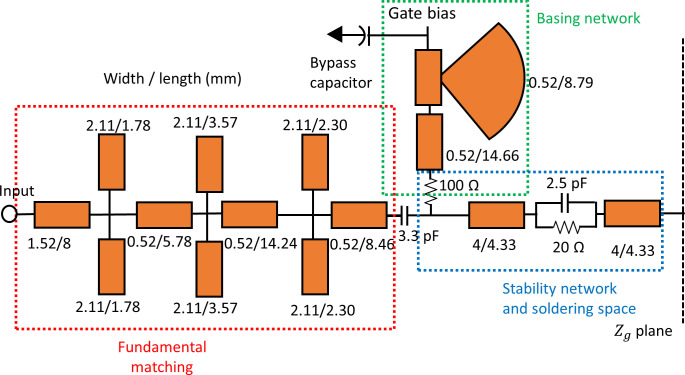
The optimized input MN.

The lump components are transformed into a transmission line using Richards’ transformation. Subsequently, the transmission line is converted into a microstrip line using the LineCalc tool provided by Keysight ADS. The final optimized input MN is shown in Fig 6. The fundamental MN is indicated by the red box and the stability network is represented by the blue box.

## Results and discussions

**Fig 7:**
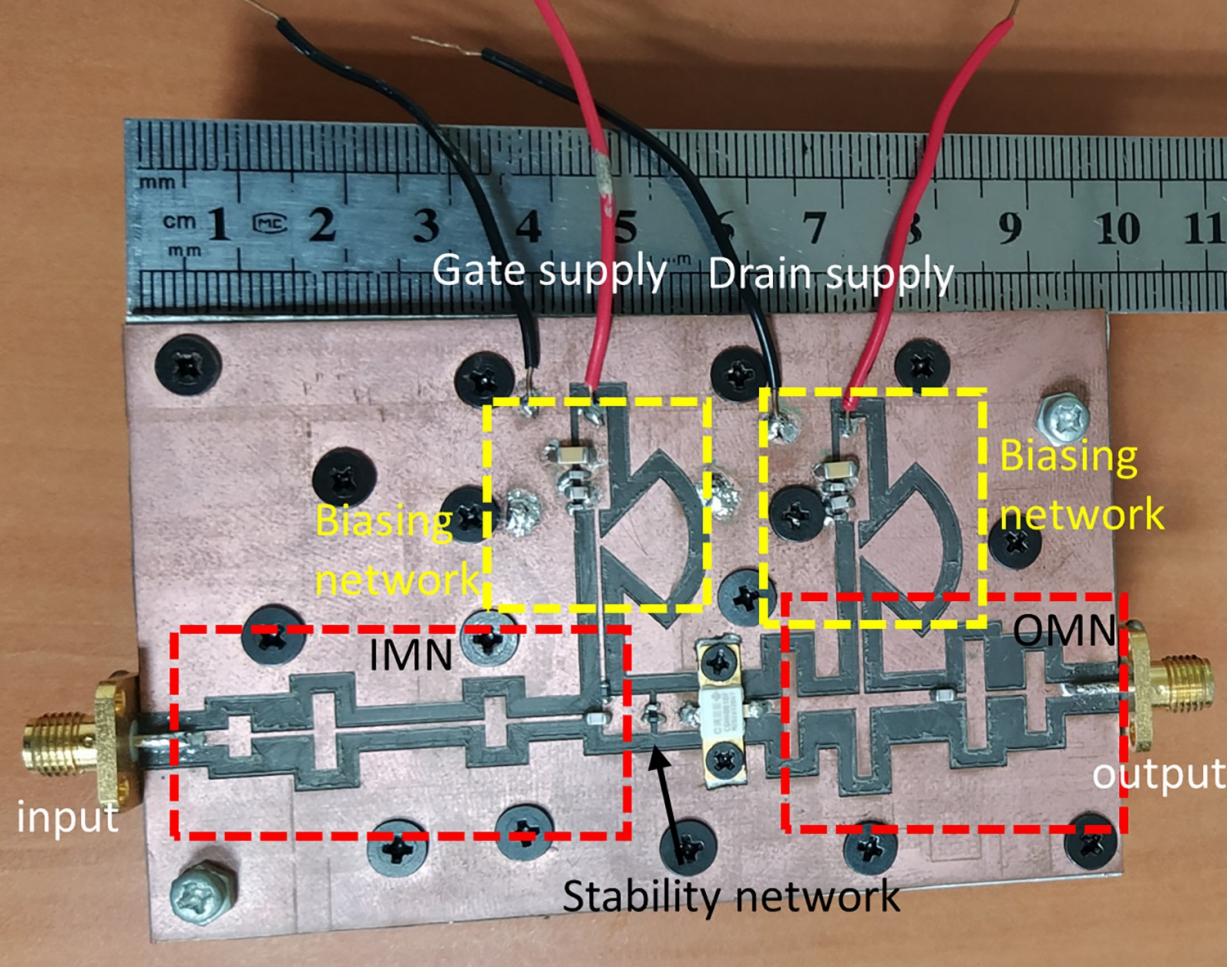
Photo of the realized CCF RFPA (size: 99 x 60 *mm*^2^).

**Fig 8:**
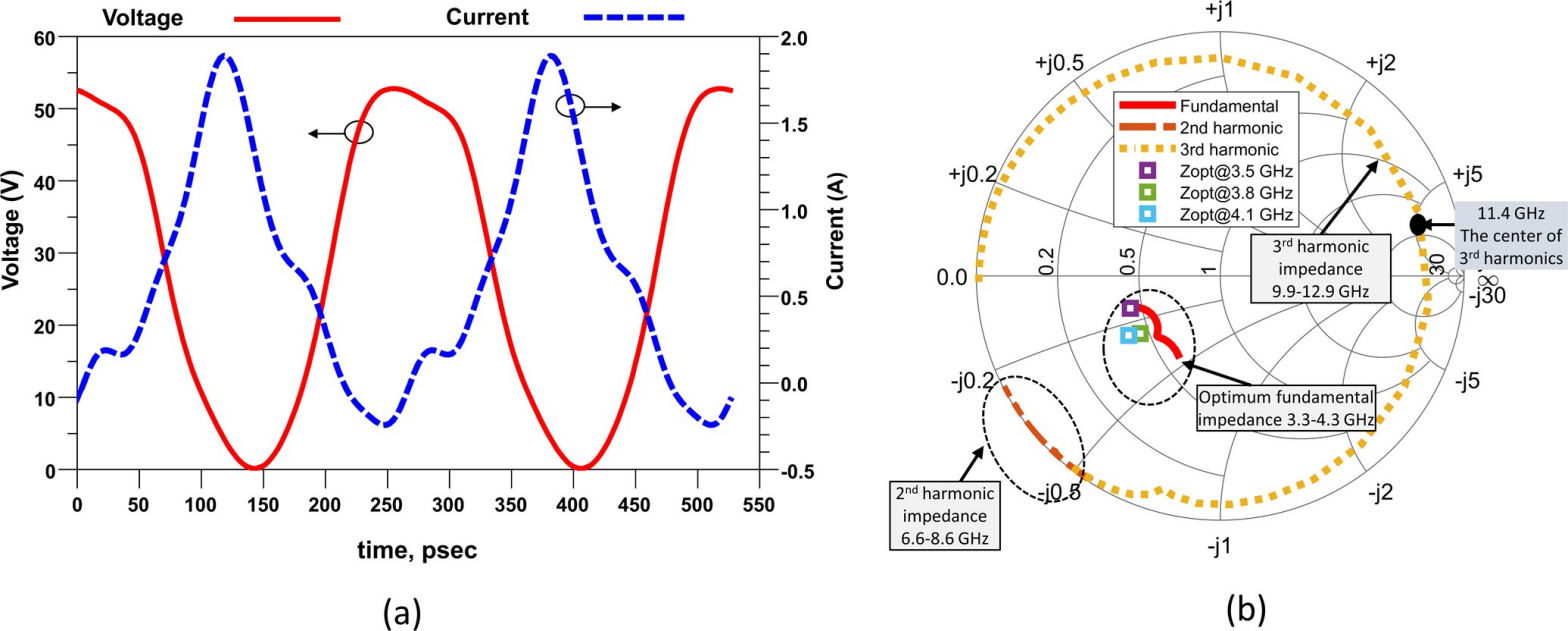
(a) Simulated voltage and current waveform at the I-Gen plane at 3.8 GHz frequency and (b) Impedance trajectories of the OMN at I-Gen plane.

Following the completion of the design, the physical implementation of the CCF mode RFPA takes place. The fabricated RFPA is depicted in Fig 7, with different colored boxes indicating the various parts of the RFPA. The prototype shown in Fig 7 comprises primarily six blocks. The drain biasing and gate biasing networks (which consist of different valued capacitors and microstrip lines) are denoted by yellow boxes on the left and right sides, respectively. The input matching network and output matching network are displayed on the left and right sides, respectively. In the center of the prototype, there are two main components: a stability network comprised of a capacitor and a resistor, indicated by an arrow, and a Cree device, distinguished by its white color. To validate the operating mode of the proposed RFPA, the harmonic-balance simulator in Keysight ADS is utilized to analyze the simulated voltage waveform at the I-Gen plane. Fig 8(A) illustrates the simulated voltage and current waveforms at the I-Gen plane when the RFPA operates at 3.8 GHz, the band’s center frequency. The voltage waveform exhibits an approximate square shape, while the current waveform appears as a half-rectified sinusoidal waveform. This observation confirms the operating mode of the CCF RFPA.

Based on the analysis of continuous class-F mode RFPA in section 3.1, harmonics need to be controlled to confirm this mode. In this design, to control the harmonics impedances up to the third, the HTN is designed as mentioned in section 3. The synthesized OMN load trajectories corresponding to the optimized OMN and the optimum load impedance of the Cree device for three frequencies (3.5 GHz, 3.8 GHz, and 4.1 GHz) at the I-Gen plane are plotted on Smith chart, as shown in Fig 8(B). It is seen that the fundamental frequency is matched for a significantly wide bandwidth. The second harmonics and third harmonics impedances are located at the low impedance and the high impedance regions of the Smith chart, respectively. The result shows that the OMN performs well for a wide bandwidth on the proposed design and this design maintains the desired impedance for the continuous class-F mode.

### Small signal S-parameter measurement

**Fig 9:**
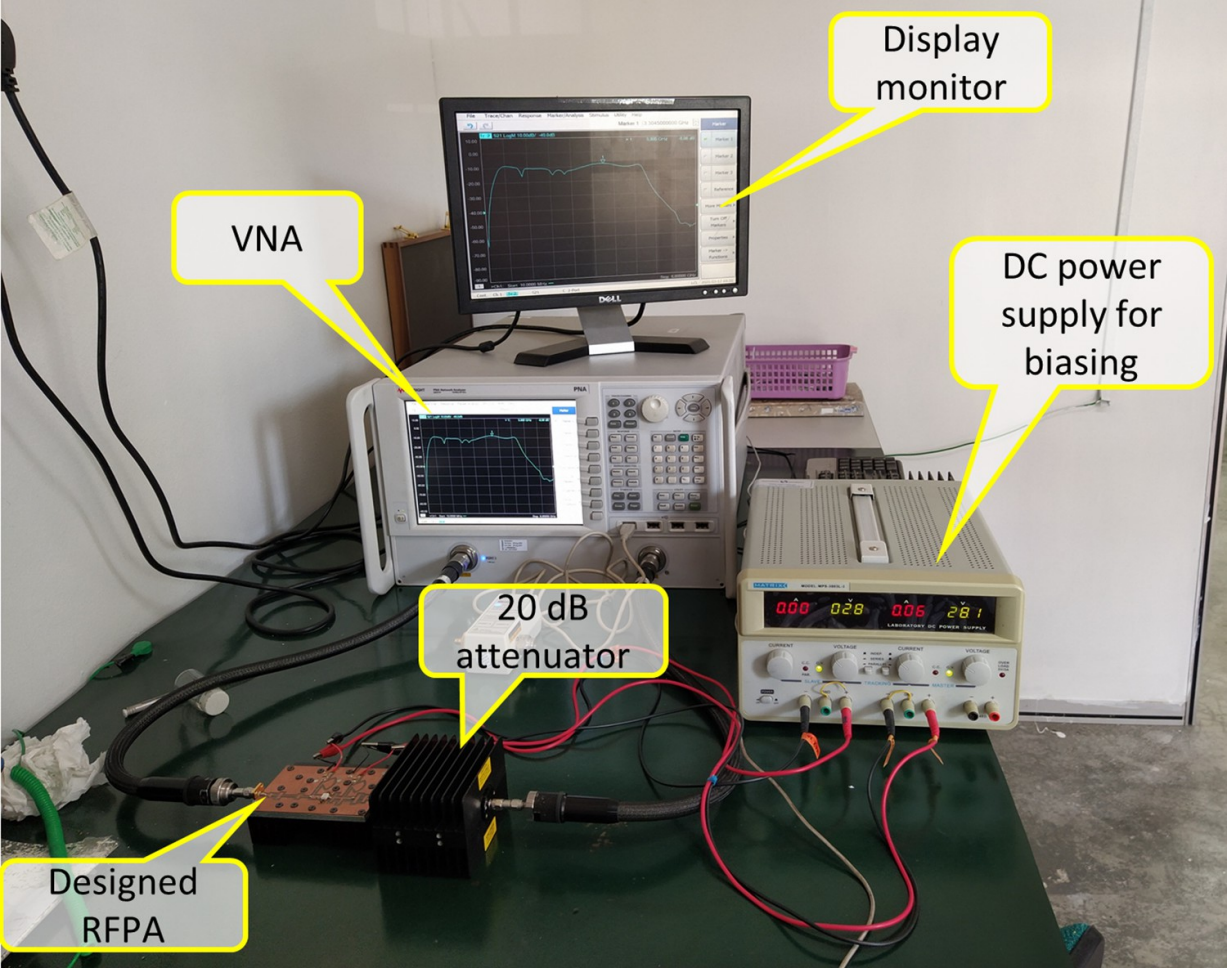
S-parameter measurement setup.

**Fig 10:**
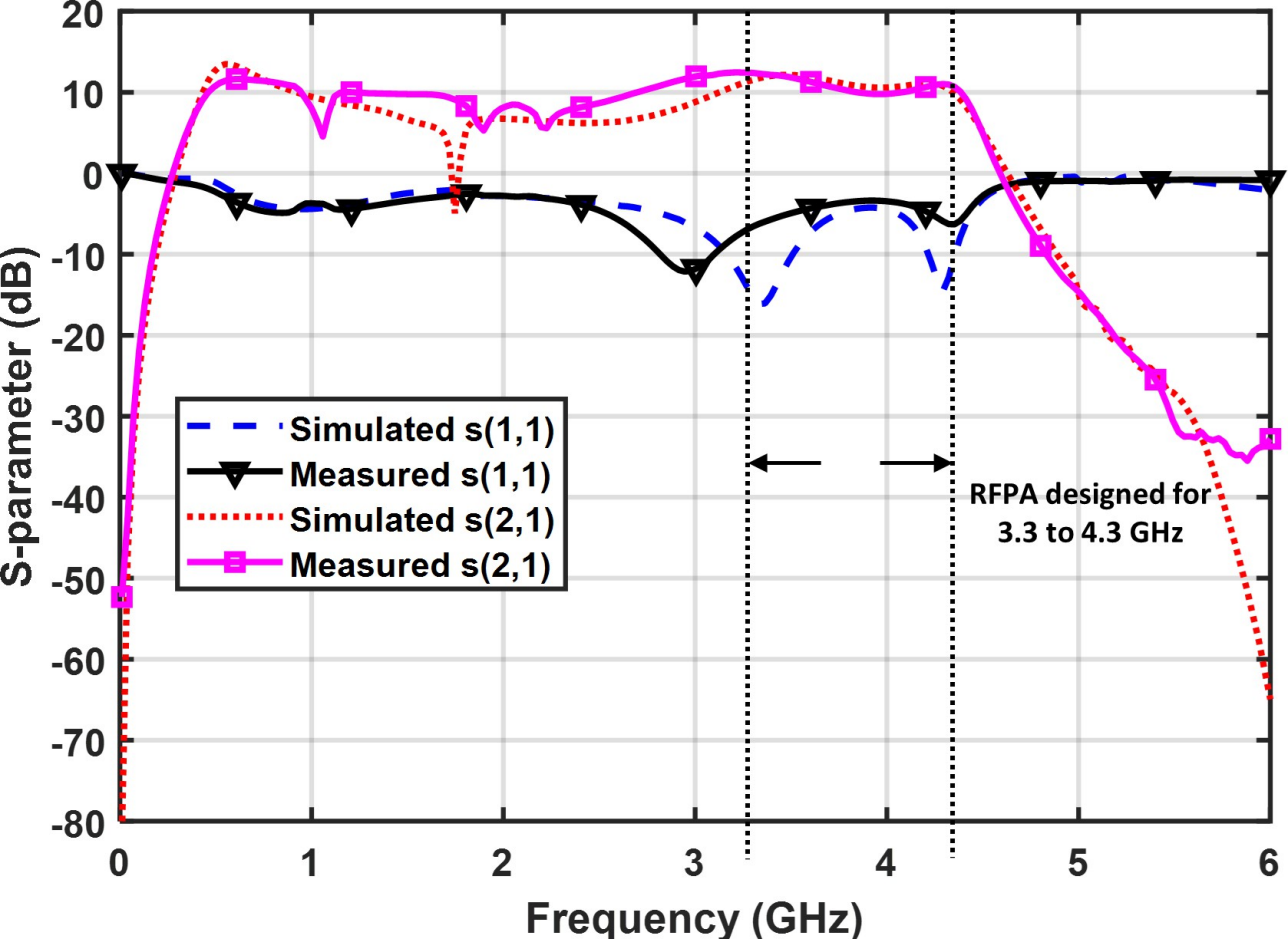
Comparison of simulated and measured S-parameter (S_11_, S_21_).

The small-signal scattering parameters are measured utilizing the Agilent PNA N5227A. To conduct a comprehensive measurement from 10 MHz to 6 GHz, the instrument is configured accordingly using an Agilent N4694-6001 electronic calibration module. Fig 9 depicts the S-parameter measuring setup. The simulated and measured small signal S-parameter comparison is shown in Fig 10. The results indicate a close comparison between the simulated and measured figures for S11 and S21 parameters. Specifically, the measured small-signal gain (S21) ranges from 12.3 dB to 9.8 dB within the frequency range of 3.3 GHz to 4.3 GHz From Fig 10, it is observed that the performance of S11 seems to be like a dual band or narrow band at two frequencies; 3.0 and 4.3 GHz which are at approximately -11 dB and -6 dB respectively as compared to the performance of the S11 at other frequencies all over the band. Although the S11 showed performance like a dual band design or narrowband at two frequencies, the S11 of the PA is still acceptable for an industry operation in the frequency band of interest. Anyway, the performance of the S11 should be improved by optimizing the input matching network for all over the band.

### Continuous wave (CW) signal measurement

**Fig 11:**
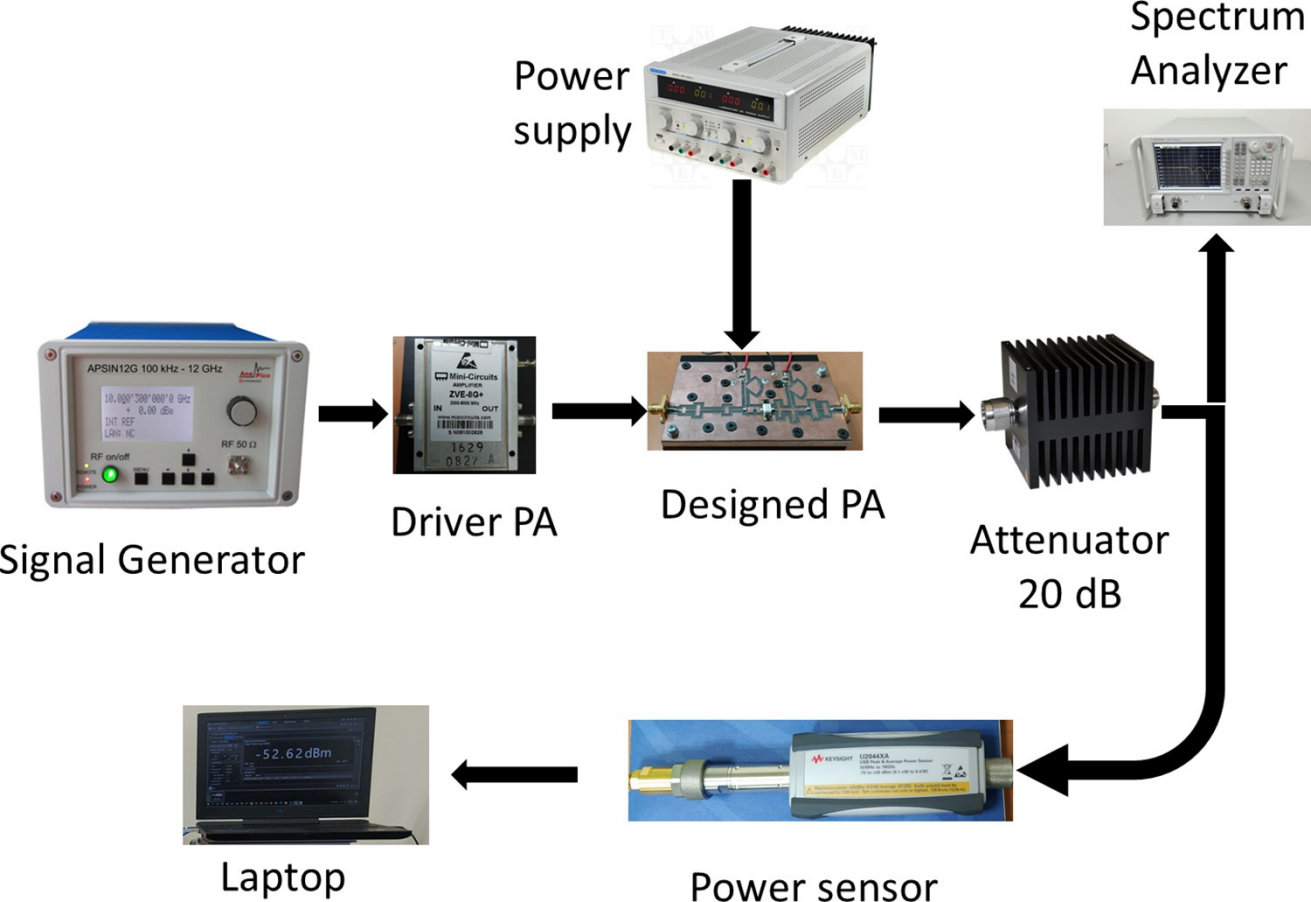
The setup for CW signal measurement.

**Fig 12:**
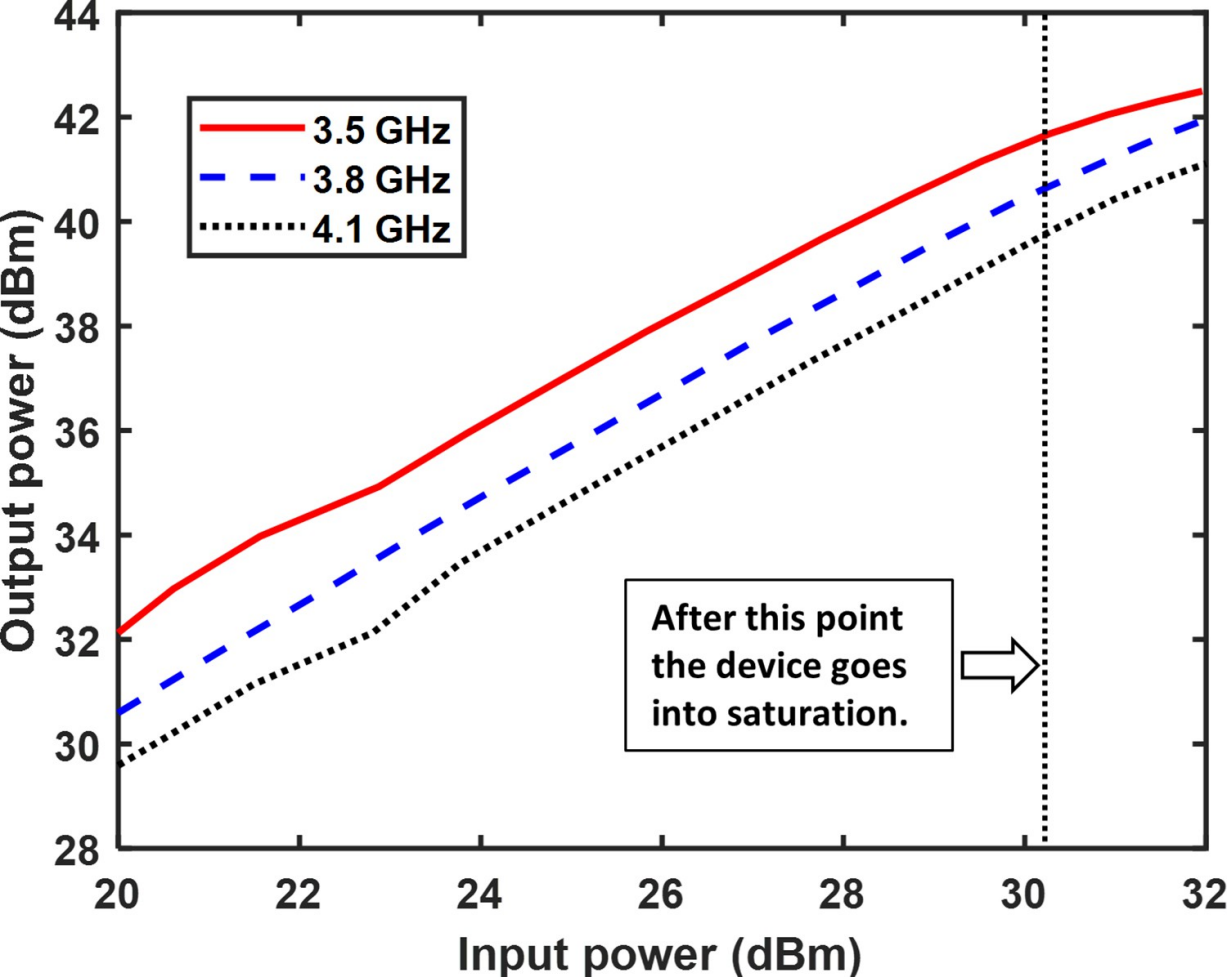
The relationship between measured output power and input power is examined at frequencies of 3.5 GHz, 3.8 GHz, and 4.1 GHz.

**Fig 13:**
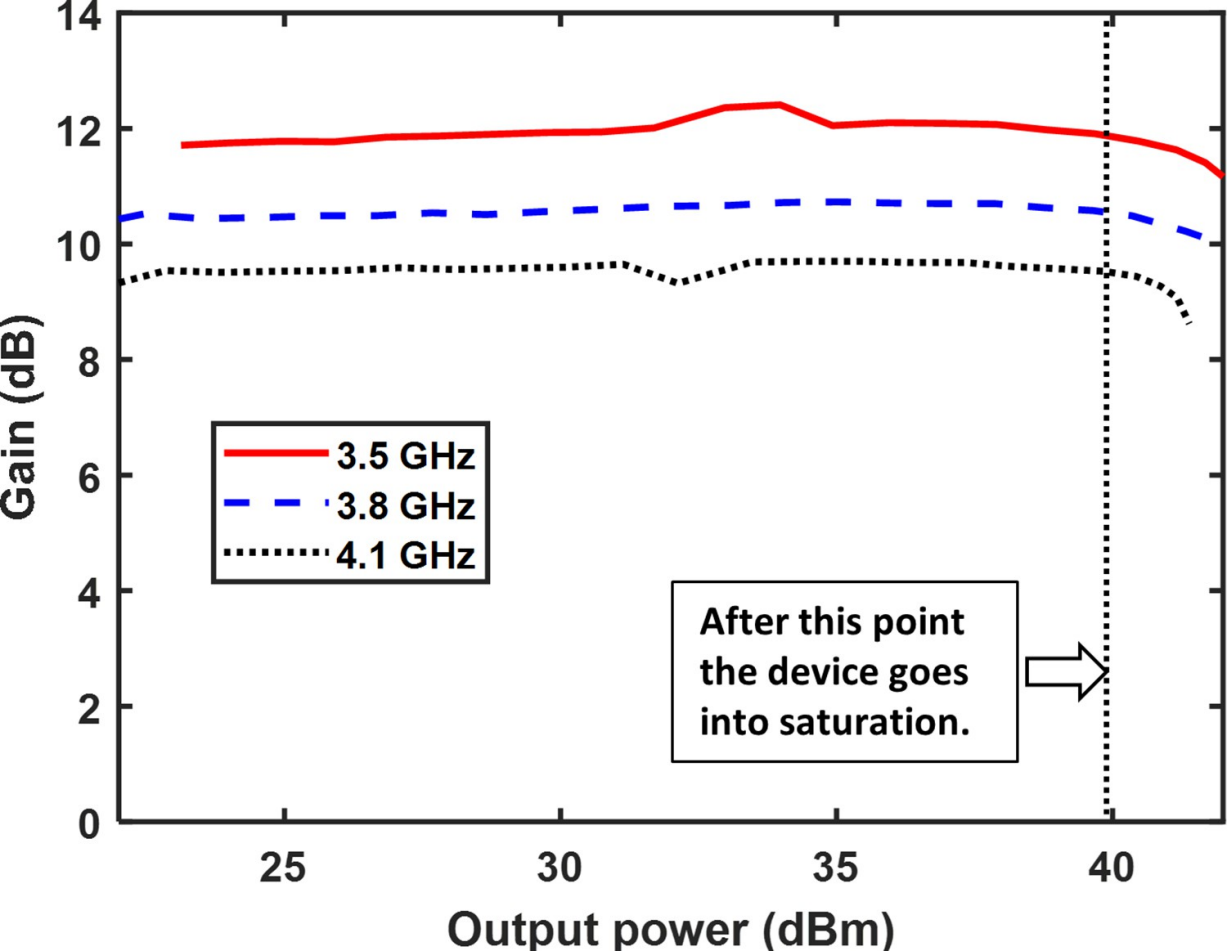
The measured gain is plotted against the output power.

Continuous wave (CW) excitation is used to evaluate the RFPA at frequencies ranging from 3.3 GHz to 4.3 GHz. Fig 11 depicts the setup utilized for the performance measurement of the CW signal. The output power is measured at three different frequencies, namely 3.3 GHz, 3.8 GHz, and 4.1 GHz, for various input power levels. The results are shown in Fig 12. In this figure, it is demonstrated that as the input power increased, the output power also increased. However, beyond input powers of 30 dBm, the output power did not exhibit a linear growth due to the saturation of the 10 W GaN Cree device. The measured gain versus output power at three frequencies is illustrated in Fig 13. The plot reveals a consistent gain as the output power increases. However, when the output power surpasses 40 dB, the gain experiences a sharp decline due to the saturation of the 10 W GaN device, which occurs beyond 40 dBm output power.

**Fig 14:**
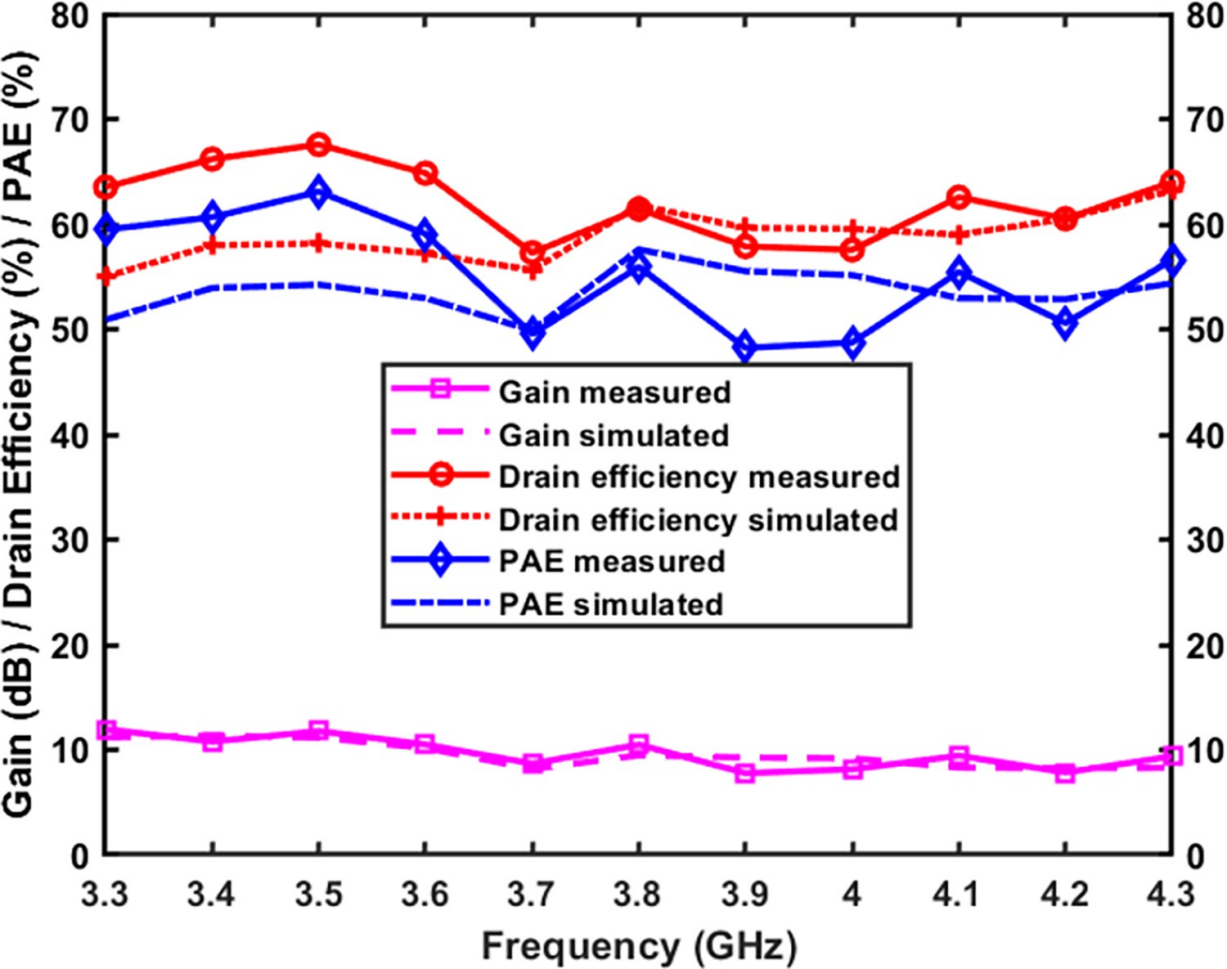
DE, PAE, and gain is plotted against the output power of 40 dBm.

Fig 14 presents the simulated and observed drain efficiency (DE), power added efficiency (PAE), gain, and output power for large signals across the frequency range of 3.3 GHz to 4.3 GHz. The measured DE at an output power of 40 dBm exceeds 57.5% throughout the operational range, with a peak value of 67.5%. The PAE exhibits a similar trend as the DE, but due to the consideration of input RF power in its calculation, the PAE tends to have a lower value than the DE. The minimum PAE is 49% at 3.9 GHz and the maximum is 63% at 3.5 GHz. The gain exceeds 8.2 dB over the entire frequency range, peaking at 11.5 dB at 3.5 GHz.

**Fig 15:**
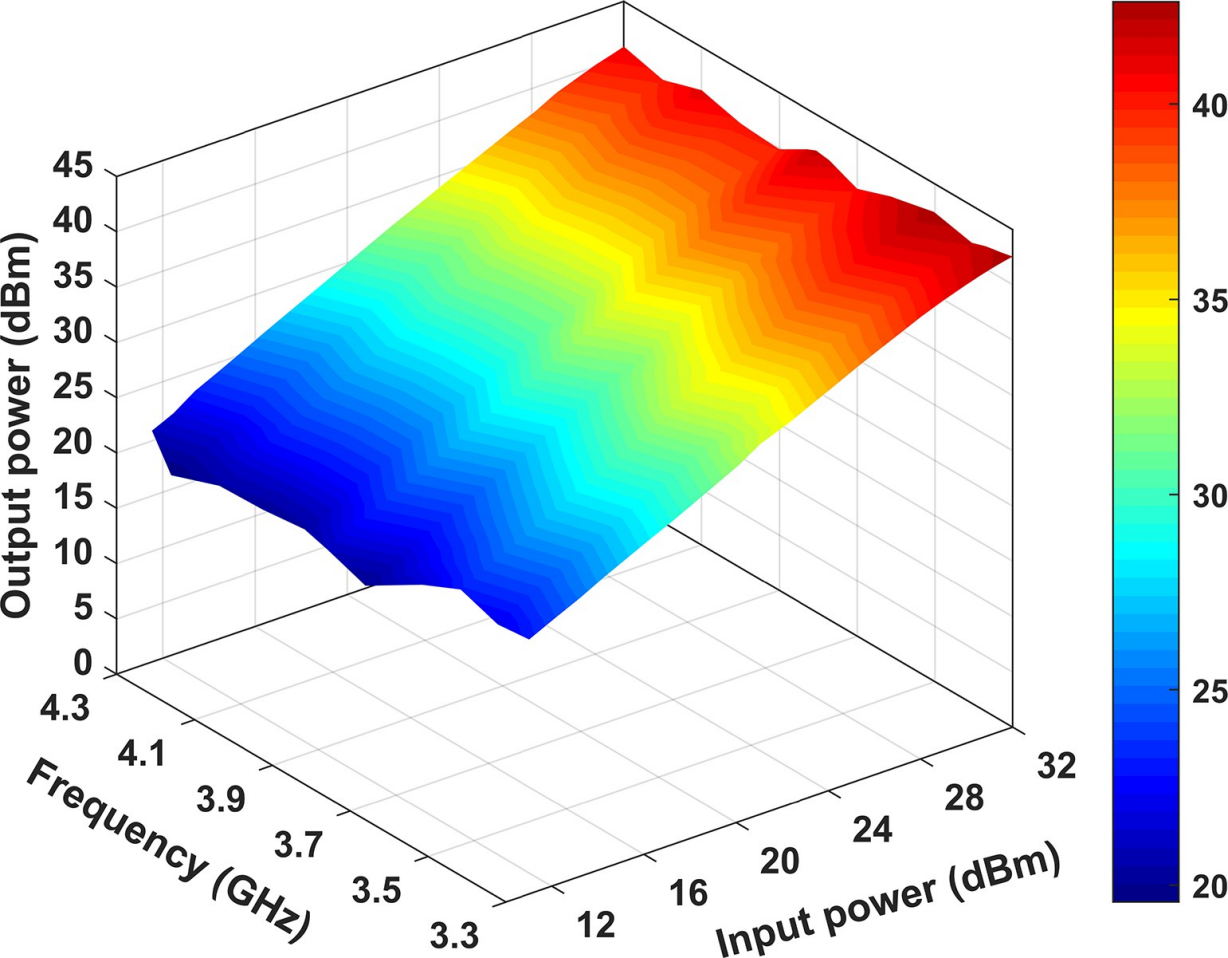
Output power is plotted against different input powers and frequencies.

**Fig 16:**
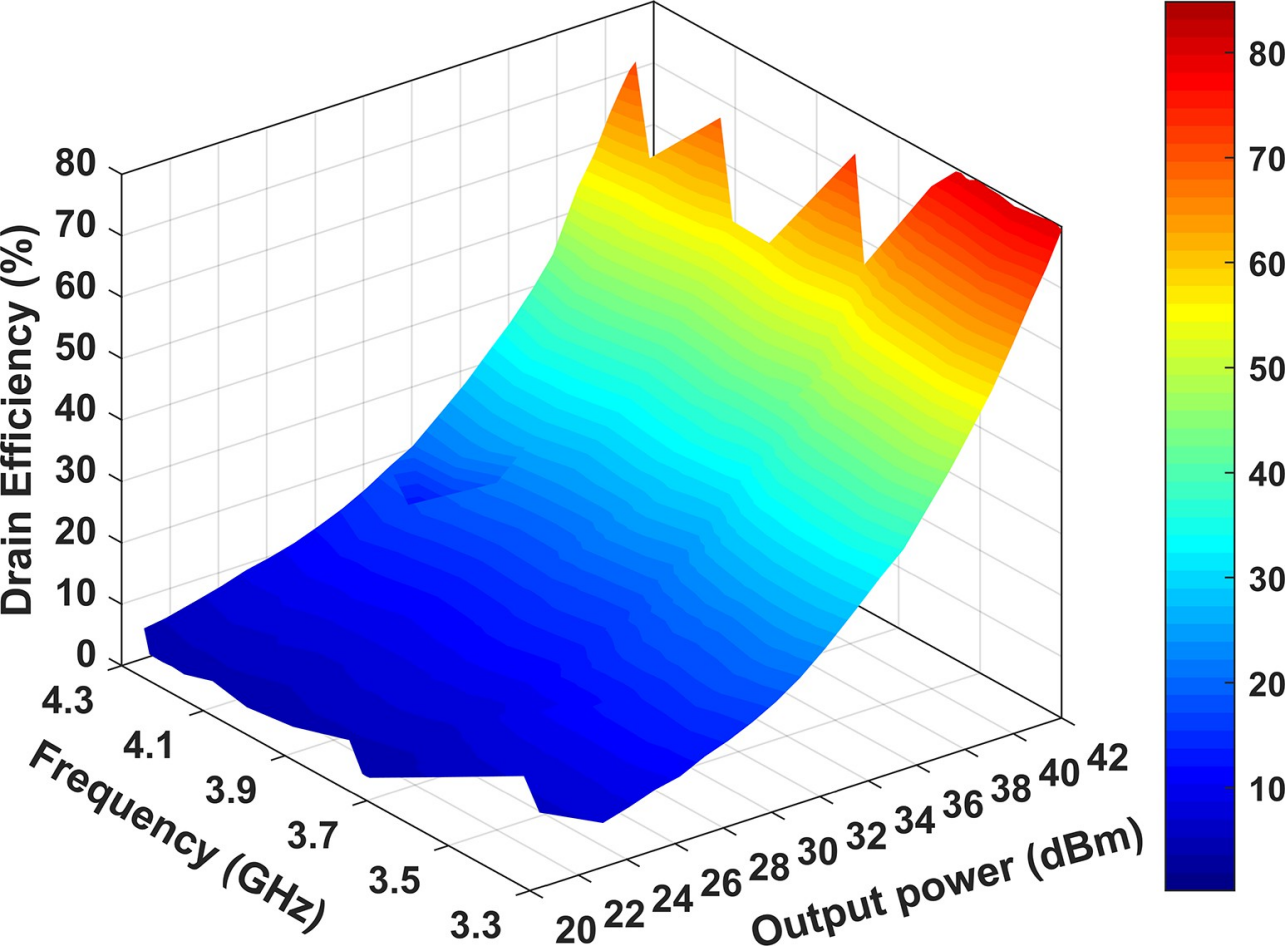
DE at different output powers and frequencies.

Fig 15 illustrates the measured performance of the designed RFPA across a range of input power from 12 to 32 dBm and frequencies spanning 3.3 GHz to 4.3 GHz. It can be observed that the output power remains above 40 dBm when the input power reaches 32 dBm across the entire frequency band. By observing Fig 16, it becomes evident that the measured peak efficiency reaches 81.2% at 3.3 GHz and 80.6% at 3.5 GHz. These efficiencies correspond to the provided output power levels of 42.2 dBm and 42 dBm, respectively. It is worth noting that these output power values exceed the nominal output power rating of the 10 W Cree device, which is set at 40 dBm. With an increase in frequency, the efficiency is decreased as the output power does not go as high as the lower frequency, and the minimum efficiency is 57.6% at 4.0 GHz at 40.2 dBm output power.

**Fig 17:**
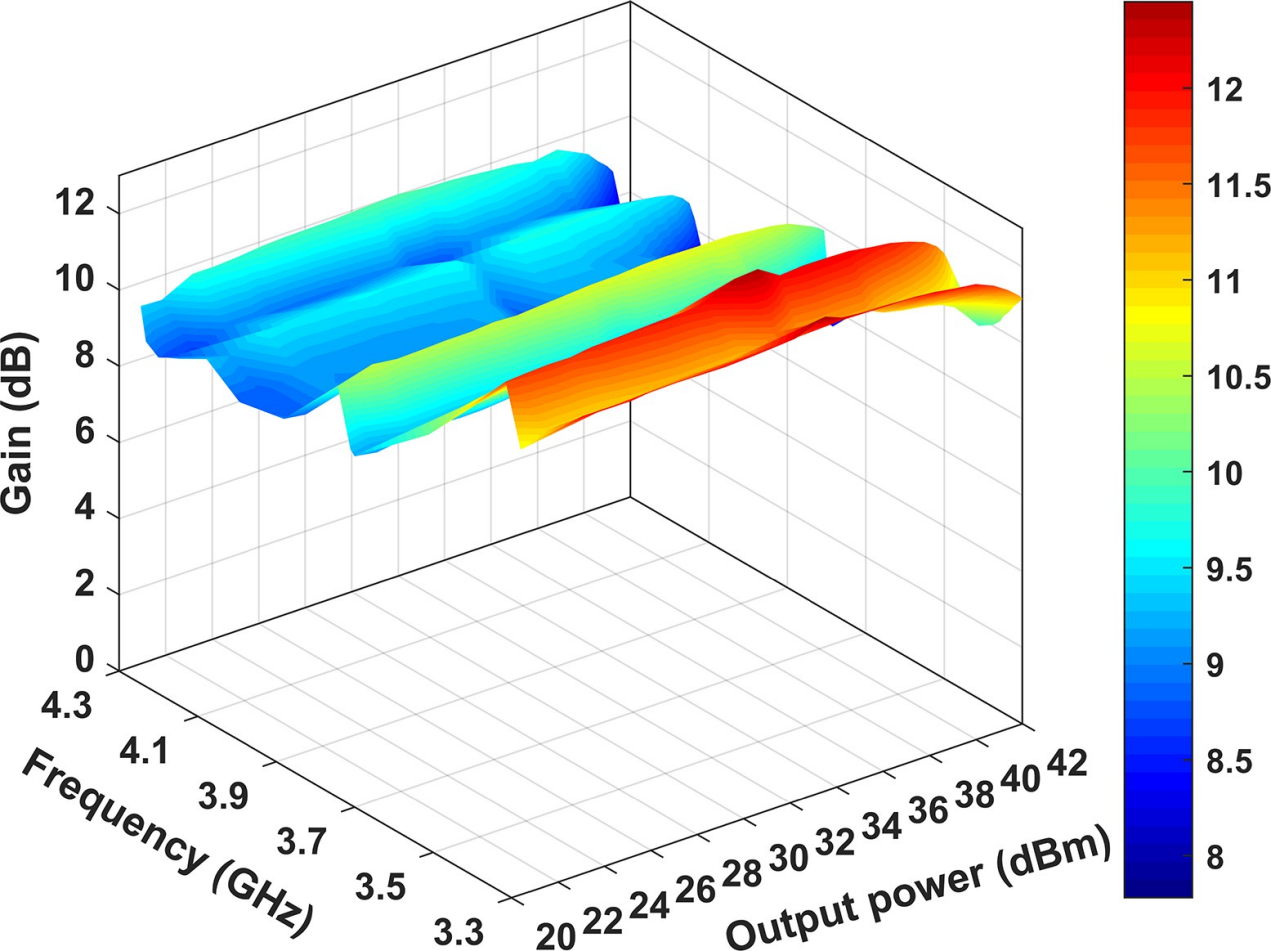
Gain at various output powers and frequencies.

Fig 17 reveals that as the frequency increases, the gain decreases. The maximum gain of 12.4 dB is achieved at 3.5 GHz, while the minimum gain of 8.4 dB is observed at 4.3 GHz. At the one dB compression point, the RFPA’s output power fluctuates between 39.2 dBm and 42.2 dBm, accompanied by a drain efficiency ranging from 52.6% to 81.2%.

### Two-tone signal measurement

**Fig 18:**
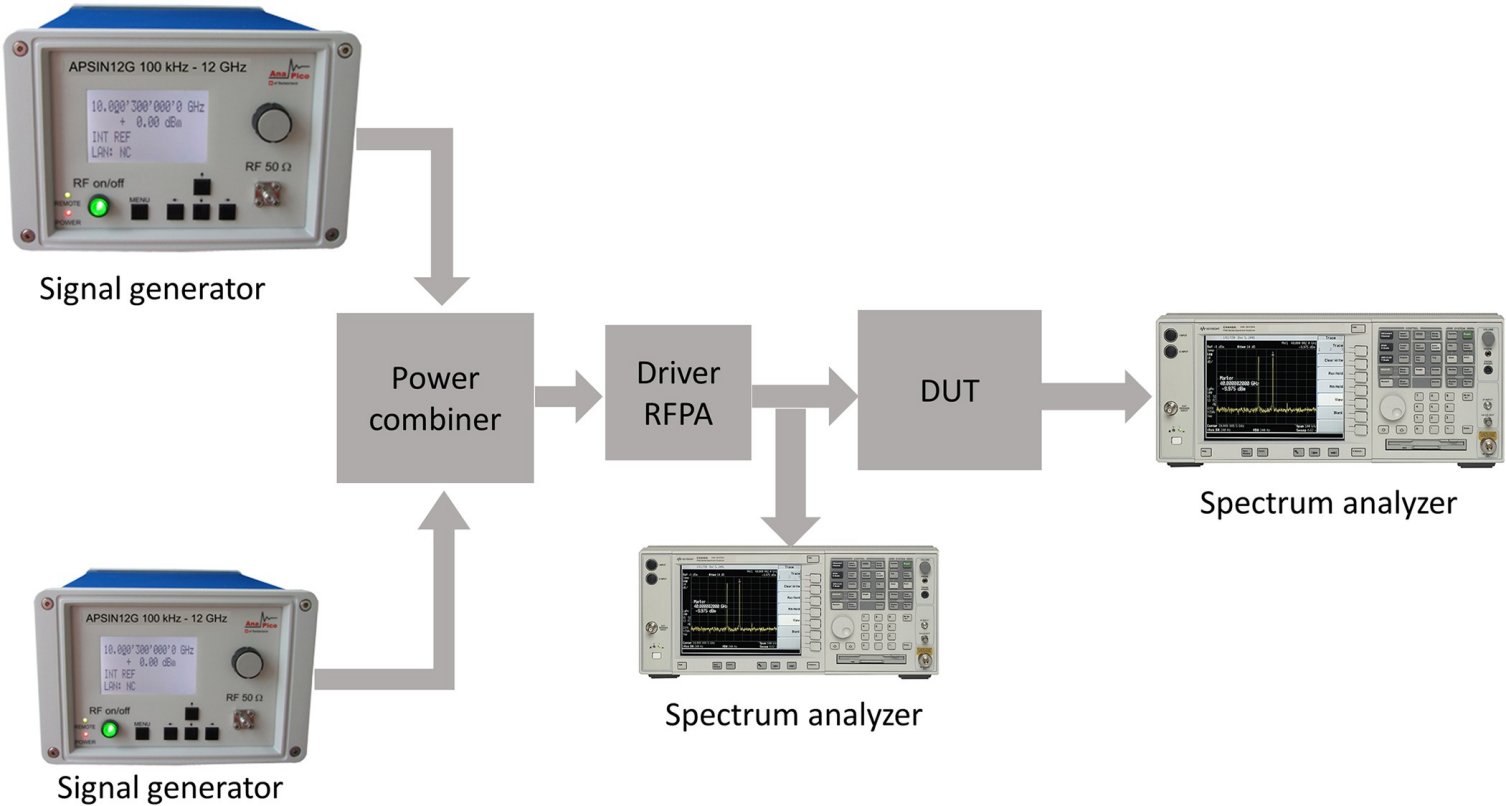
IMD3 measurement setup.

**Fig 19:**
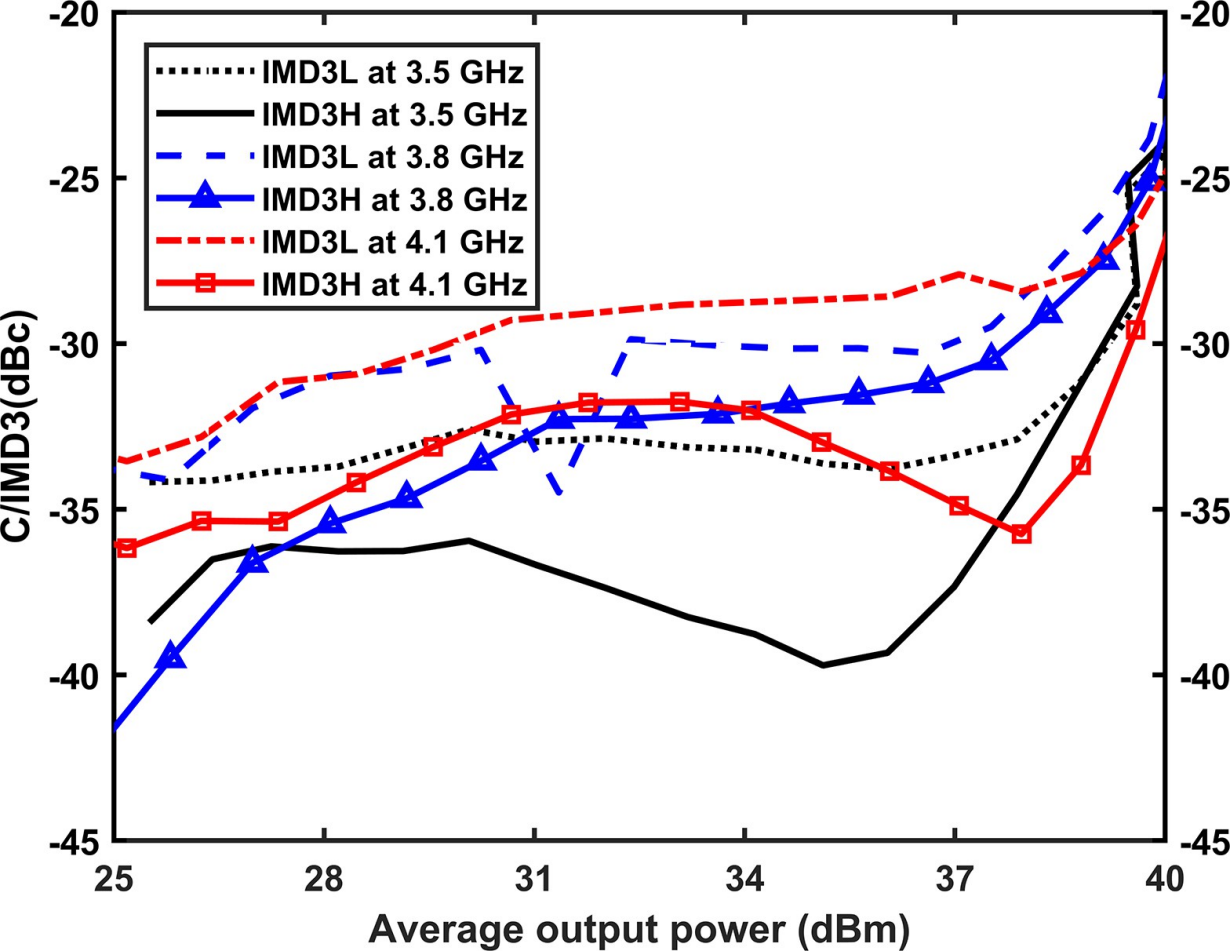
C/IMD3 is plotted against the average output power at 3.5 GHz, 3.8 GHz and 4.1 GHz.

To assess the linearity of the implemented RFPA, a two-tone signal with a frequency spacing of 10 MHz is employed for testing at specific frequency centers. The testing is conducted at the center of the lower band (3.5 GHz), the center of the entire band (3.8 GHz), and the center of the upper band (4.1 GHz). The measurement configuration for the two-tone testing is depicted in Fig 18. The correlation between the average output power and the carrier to third-order intermodulation distortion (C/IMD3) ratio is presented in Fig 19. Throughout the entire frequency band, the C/IMD3 ratio remains lower than -22 dBc for average output powers up to 40 dBm.

**Fig 20:**
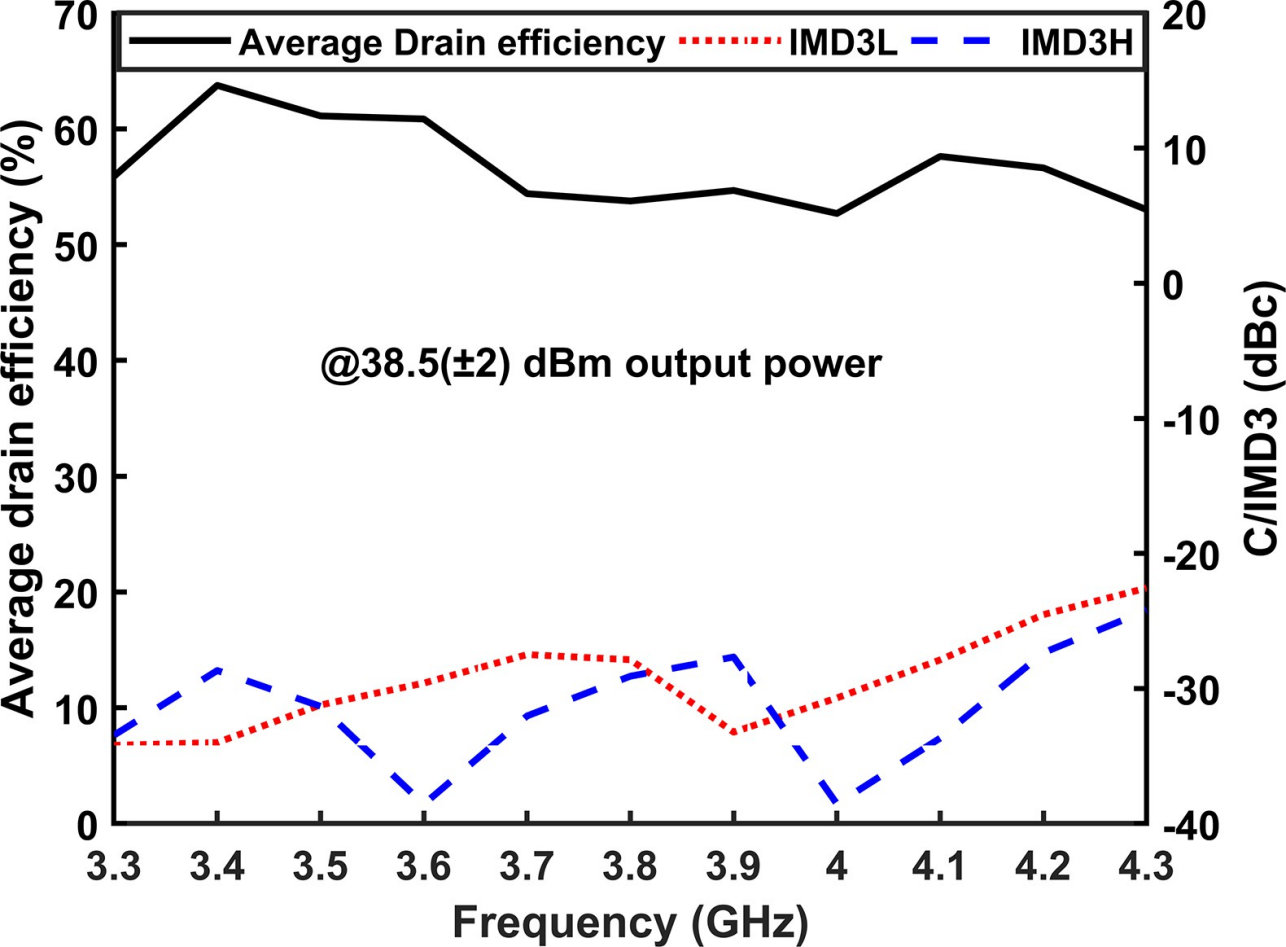
C/IMD3 and the DE are plotted at 38.5 (±0.2) dBm power against frequency.

To evaluate the linearity of the entire frequency band, a two-tone signal with a 10 MHz spacing is applied between 3.3 GHz and 4.3 GHz at an output level of 38.5 (±0.2) dBm. As shown in Fig 20, it is evident that superior linearity performance is achieved at lower frequencies compared to higher frequencies. Throughout the entire band, the linearity is maintained below -22 dBc. At an output power of 38.5 (±0.2) dBm, the average drain efficiency (DE) ranges from 53% to 63.3%, which indicates commendable performance.

Table 4 summarizes the state-of-the-art wide band RFPA of the CCF mode using the Cree 10 W GaN device found in the literature. The amplifiers mentioned in Table 4 are designed using different matching techniques. The overall performance of the designed RFPA is comparable with that of other RFPAs. From the CW signal measurement, it can be said that the designed RFPA, which has applied the SRFT technique, can perform better at the 5G sub 6 GHz band as compared to other RFPAs in terms of the stable output power and operating frequencies. Most of the RFPAs listed in Table 4 are also not suitable for 5G because the operating frequencies shown are not in the 5G frequency band range. The designed RFPA has a one GHz bandwidth and operates at the highest frequencies (3.3–4.3 GHz) among the RFPAs in Table 4 with comparable DE and output power.

**Table 4:** Recent research on wide band RFPAs.

Ref.	Year	Frequency	Matching	Class of	Output power (dBm)	Gain (dB)	DE (%)
		(GHz)	Techniques	RFPA			
[16]	2017	0.4–2.3	SRFT	CCF	39–42	11–14	62.3–80.5
[17]	2017	1.4–2.4	BPF & SRFT	CCF	>40	9.5–12.7	60–73
[18]	2018	1.2–3.6	N/M	CCF	40–42.2	10.5–12.5	60–72
[19]	2019	1.7–3.0	BPM	CCF	39.7–41.1	9.7–11	54.7–74.8
[20]	2020	1.35–2.35	MSHCMN	CCF-1	40.1–41.5	10–11.5	71–82
[21]	2021	0.2–1.7	CLPF	ECCF	38–40.2	13–15.2	53–79
[22]	2022	1.5–3.0	CLPF	CCF-1	40.2–42.2	12.2–14.2	65–77
[23]	2023	1.8–2.5	HMM	AB	38.4–39.7	14	PAE>50%
This work	2023	3.3–4.3	SRFT	CCF	40 (±0.3)	8.4–12.4	57.5–67.5

CCF- Continuous Class-F, ECCF–Extended Continuous Class- F, CCF-1 Inverse Continuous Class-F, SRFT-Simplified Real Frequency Technique, BPF- Band Pass Filter, BPM- Band Pass Matching, N/M- Not Mentioned, MSHCMN- Multistage Second Harmonic Control Matching Network, CLPF- Chebyshev Low Pass Filter. HMM-Hidden Markov Model.

## Conclusions

This study applies the CCF mode RFPA design approach to the 5G frequency spectrum. The design ensures high efficiency across a wide bandwidth by carefully managing the impedance of both the fundamental and harmonic frequencies at the I-Gen plane. The matching networks corresponding to the optimal fundamental and harmonic frequencies are separately designed using the SRFT algorithm and other complementary methodologies, simplifying the design process. The measured DE at the 40 dBm output power is between 57.5% to 67.5% throughout the frequency band of 3.3 GHz to 4.3 GHz. Also, when measured at one dB compression point, where this point can be more than 40dBm, the constructed RFPA achieved the DE of 52.6% to 81.2% across the same frequency range. Additionally, the linearity performance of the RFPA is evaluated using a two-tone signal with a 10 MHz spacing, revealing linearity performance below -22 dBc across the entire band.

## Supporting information

S1 Fig n^th^ order low pass structure. (TIF)

S2 Fig Normalized MN from the active device to reference impedance. (TIF)

S3 Fig Load-pull and source-pull setup. (TIF)

S4 Fig The optimized output MN. (TIF)

S5 Fig TPG versus normalized frequency. (TIF)

S6 Fig The optimized input MN. (TIF)

S7 Fig Photo of the realized CCF RFPA (size: 99 x 60 *mm*^2^). (TIF)

S8 Fig (a) Simulated voltage and current waveform at the I-Gen plane at 3.8 GHz frequency and (b) Impedance trajectories of the OMN at I-Gen plane.
(TIF)

S9 Fig S-parameter measurement setup. (TIF)

S10 Fig Comparison of simulated and measured S-parameter (S11, S21). (TIF)

S11 Fig The setup for CW signal measurement. (TIF)

S12 Fig The relationship between measured output power and input power is examined at frequencies of 3.5 GHz, 3.8 GHz, and 4.1 GHz. (TIF)

S13 Fig The measured gain is plotted against the output power. (TIF)

S14 Fig DE, PAE, and gain is plotted against the output power of 40 dBm. (TIF)

S15 Fig Output power is plotted against different input powers and frequencies. (TIF)

S16 Fig DE at different output powers and frequencies. (TIF)

S17 Fig Gain at various output powers and frequencies. (TIF)

S18 Fig IMD3 measurement setup. (TIF)

S19 Fig C/IMD3 is plotted against the average output power at 3.5 GHz, 3.8 GHz and 4.1 GHz. (TIF)

S20 Fig C/IMD3 and the DE are plotted at 38.5 (±0.2) dBm power against frequency. (TIF)

## References

[1] Ancans G., Bobrovs V., Ancans A., and Kalibatiene D., “Spectrum Considerations for 5G Mobile Communication Systems,” *Procedia Comput*. *Sci*., vol. 104, pp. 509–516, Dec. 2016, doi: 10.1016/j.procs.2017.01.166

[2] Sadeque M. G., Yusoff Z., and Roslee M., “A High-Efficiency Continuous Class-F Power Amplifier Design using Simplified Real Frequency Technique,” *Bull*. *Electr*. *Eng*. *Informatics*, vol. 9, no. 5, pp. 1924–1932, Oct. 2020, doi: 10.11591/eei.v9i5.2227

[3] Yang Z., Yao Y., Liu Z., Li M., Li T., and Dai Z., “Design of High Efficiency Broadband Continuous Class-F Power Amplifier Using Real Frequency Technique with Finite Transmission Zero,” *IEEE Access*, vol. 6, pp. 61983–61993, Oct. 2018, doi: 10.1109/ACCESS.2018.2875010

[4] Cripps S. C., Tasker P. J., Clarke A. L., Lees J., and Benedikt J., “On the Continuity of High Efficiency Modes in Linear RF Power Amplifiers,” *IEEE Microw*. *Wirel*. *Components Lett*., vol. 19, no. 10, pp. 665–667, Oct. 2009, doi: 10.1109/LMWC.2009.2029754

[5] Cripps S. C., *RF Power Amplifiers for Wireless Communications*, Second., vol. 53, no. 9. 1981.

[6] Carlin H. J. and Komiak J. J., “A New Method of Broadband Equalization Applied to Microwave Amplifiers,” *IEEE Trans*. *Microw*. *Theory Tech*., vol. 27, no. 2, pp. 93–99, 1979, doi: 10.1109/TMTT.1979.1129569

[7] Yarman B. S., “A Simplified ‘Real Frequency’ Technique Appliable To Broadband Multistage Microwave Amplifiers,” in *IEEE MTT-S Digest*, 1982, vol. MIT-30, no. 12, pp. 529–531. doi: 10.1109/TMTT.1982.1131411

[8] Yarman B. S., “Design of Ultra-Wideband Antenna Matching Networks,” *IEEE Appl*. *Electromagn*. *Conf*., pp. 1–4, 2007.

[9] Sadeque M. G., Yusoff Z., and Roslee M., “Design of Wideband Continuous Class-F Power Amplifier Using Low Pass Matching Technique and Harmonic Tuning Network,” *IEEE Access*, vol. PP, p. 1, Sep. 2022, doi: 10.1109/ACCESS.2022.3202886

[10] Yarman D. B. S., *Design of Ultra Wideband Antenna Matching Networks Via Simplified Real Frequency Technique*. Springer, 2008.

[11] An H., Nauwelaers B. K. J. C., and Van De Capelle A. R., “Broadband Microstrip Antenna Design with the Simplified Real Frequency Technique,” vol. 42, no. 2, pp. 129–136, 1994.

[12] Yarman B. S., Retdian N., Takagi S., and Fujii N., “Gain-Bandwidth Limitations of 0.18 μ m Si-CMOS RF Technology,” in *18th European Conference on Circuit Theory and Design*, 2007, no. 1, pp. 264–267.

[13] Sadeque M. G., Yusoff Z., Roslee M., Hashim S. J., and Mohd Marzuki A. S., “Analysis and design of the biasing network for 1 GHz bandwidth RF power amplifier,” *Indones*. *J*. *Electr*. *Eng*. *Comput*. *Sci*., vol. 24, no. 1, pp. 308–316, Oct. 2021, doi: 10.11591/ijeecs.v24.i1.pp308-316

[14] Pozar David M., *Microwave Engineering*, 4th ed. John Wiley & Sons, Inc, 2012.

[15] Du X. et al., “Wideband high-efficiency linearized PA design with reduction in memory effects and IMD3,” *Int*. *J*. *Microw*. *Wirel*. *Technol*., vol. 10, no. 4, pp. 391–400, 2018, doi: 10.1017/S1759078718000417

[16] Tang Q. H., Li Y. H., and Li W. G., “Over Second Octave Power Amplifier Design Based on Resistive-Resistive Series of Continuous Class-F/F-1 Modes,” *IEEE Microw*. *Wirel*. *Components Lett*., vol. 27, no. 5, pp. 494–496, May 2017, doi: 10.1109/LMWC.2017.2690847

[17] Aggrawal E., Rawat K., and Roblin P., “Investigating Continuous Class-F Power Amplifier Using Nonlinear Embedding Model,” *IEEE Microw*. *Wirel*. *Components Lett*., vol. 27, no. 6, pp. 593–595, Jun. 2017, doi: 10.1109/LMWC.2017.2701316

[18] Huang C., He S., Shi W., and Song B., “Design of Broadband High-Efficiency Power Amplifiers Based on the Hybrid Continuous Modes with Phase Shift Parameter,” *IEEE Microw*. *Wirel*. *Components Lett*., vol. 28, no. 2, pp. 159–161, Feb. 2018, doi: 10.1109/LMWC.2017.2787061

[19] Liu G., Mu F., Qiu X., Leng Y., and Peng X., “Design of Broadband Power Amplifier Based on Continuous Class-F Mode with Frequency Parameterization,” *IEICE Electron*. *Express*, vol. 16, no. 6, pp. 1–4, Mar. 2019, doi: 10.1587/elex.16.20190038

[20] Wang T., Cheng Z., Liu G., Li S., and Zhang Z., “Highly efficient broadband continuous inverse Class-F power amplifier using multistage second harmonic control output matching network,” *Int*. *J*. *RF Microw*. *Comput*. *Eng*., vol. 30, no. 5, pp. 1–8, 2020, doi: 10.1002/mmce.22162

[21] Zarghami S., Hayati M., Kazimierczuk M. K., and Sekiya H., “A novel design methodology for extended continuous class-F power amplifiers in wireless applications,” *Wirel*. *Networks*, vol. 27, no. 6, pp. 3947–3968, 2021, doi: 10.1007/s11276-021-02718-8

[22] Xia J. et al., “Optimization design of fragment-type filtering matching network for continuous inverse class-F power amplifier,” *IEICE Electron*. *Express*, vol. 19, no. 14, pp. 1–5, 2022, doi: 10.1587/elex.19.20220043

[23] Soruri M., Razavi S. M., Forouzanfar M., and Colantonio P., “Design and fabrication of a GaN HEMT power amplifier based on hidden Markov model for wireless applications,” *PLoS One*, vol. 18, no. 5 MAY, pp. 1–18, 2023, doi: 10.1371/journal.pone.0285186 37146032 PMC10162524

