# Supplementary figures and images for: 3.3–4.3 GHz efficient continuous class-F gallium nitride power amplifier based on simplified real frequency technique and harmonic tuning
Source: PLoS One. 2024 Aug 14;19(8):e0306738. doi: 10.1371/journal.pone.0306738 (PMC11324125; doi:10.1371/journal.pone.0306738)

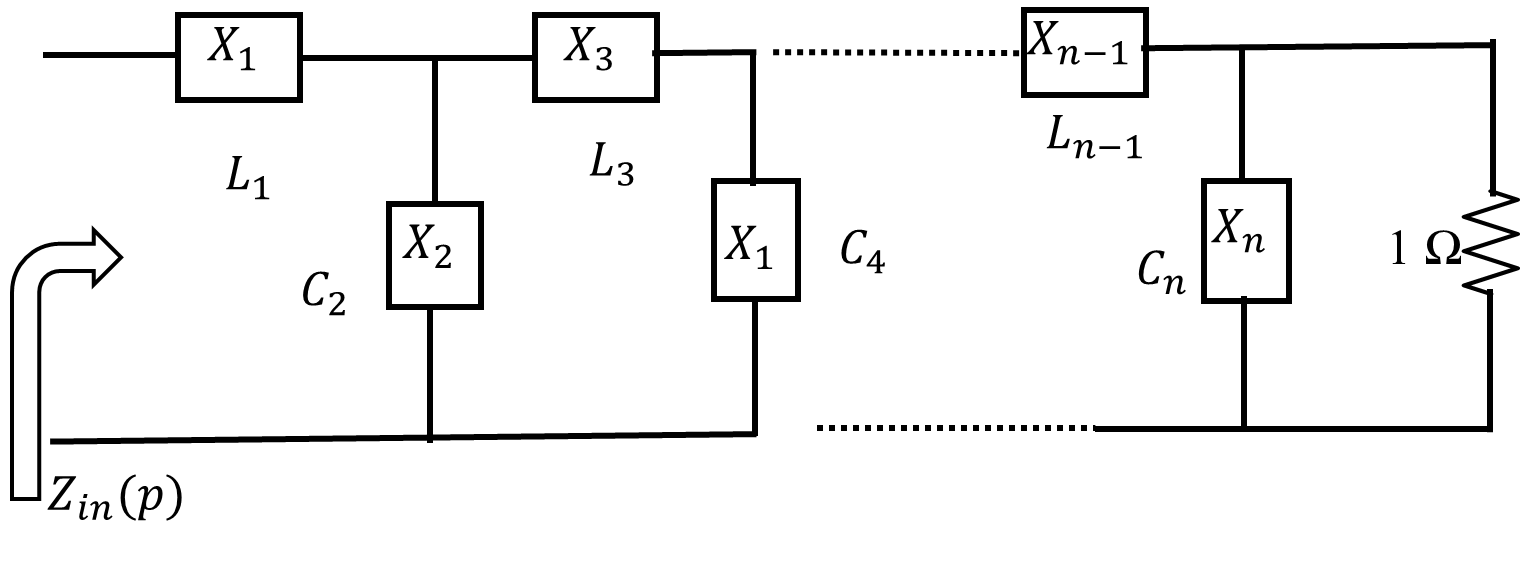

Supplement: S1 Fig — (TIF) [file pone.0306738.s001.tif]

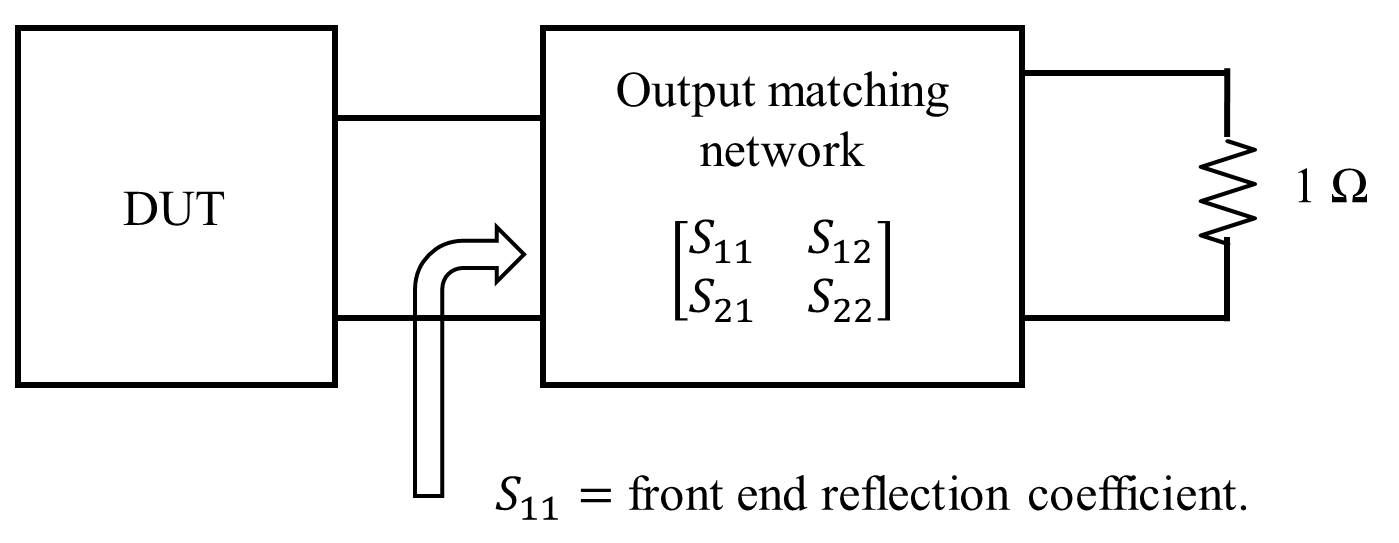

Supplement: S2 Fig — (TIF) [file pone.0306738.s002.tif]

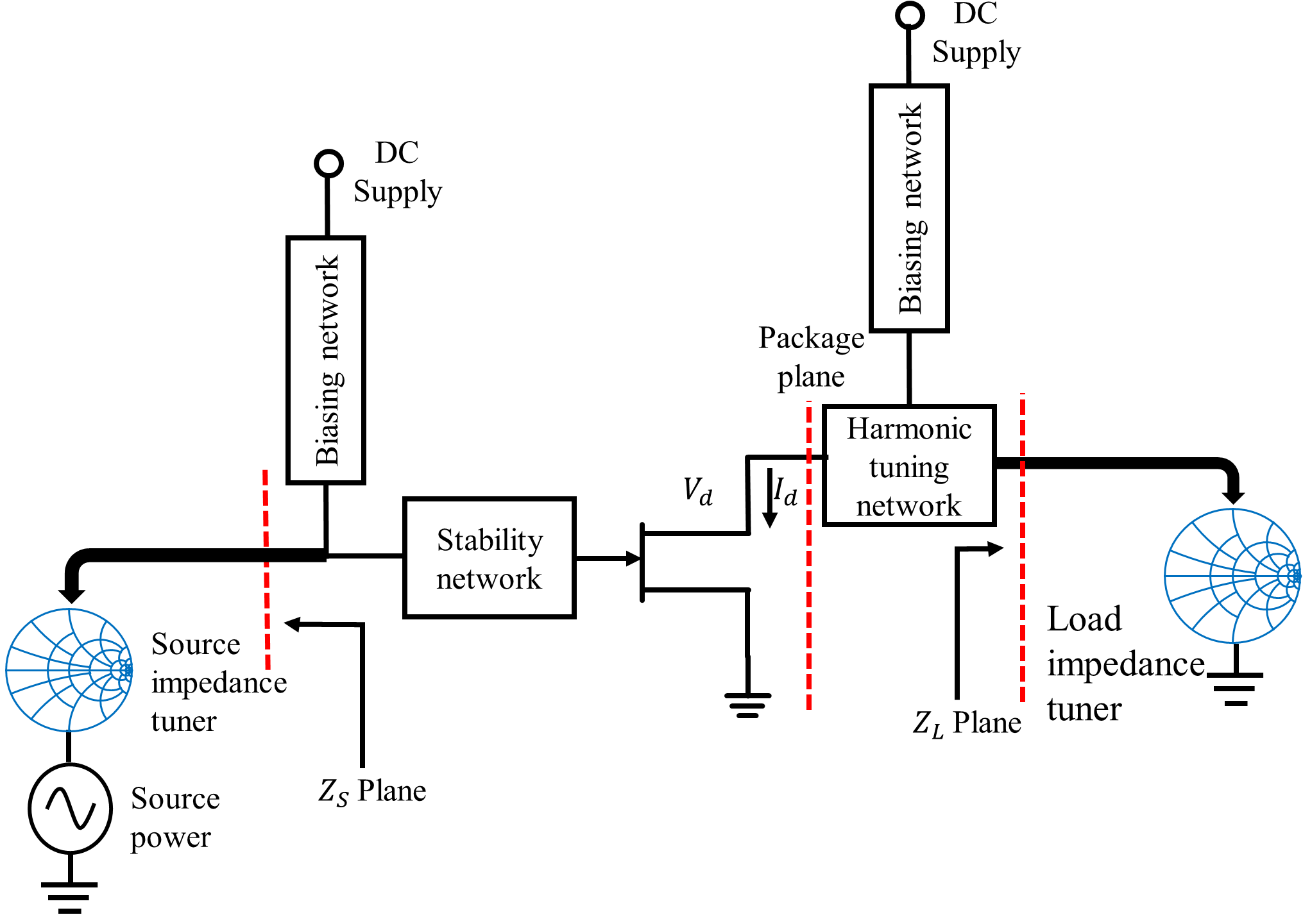

Supplement: S3 Fig — (TIF) [file pone.0306738.s003.tif]

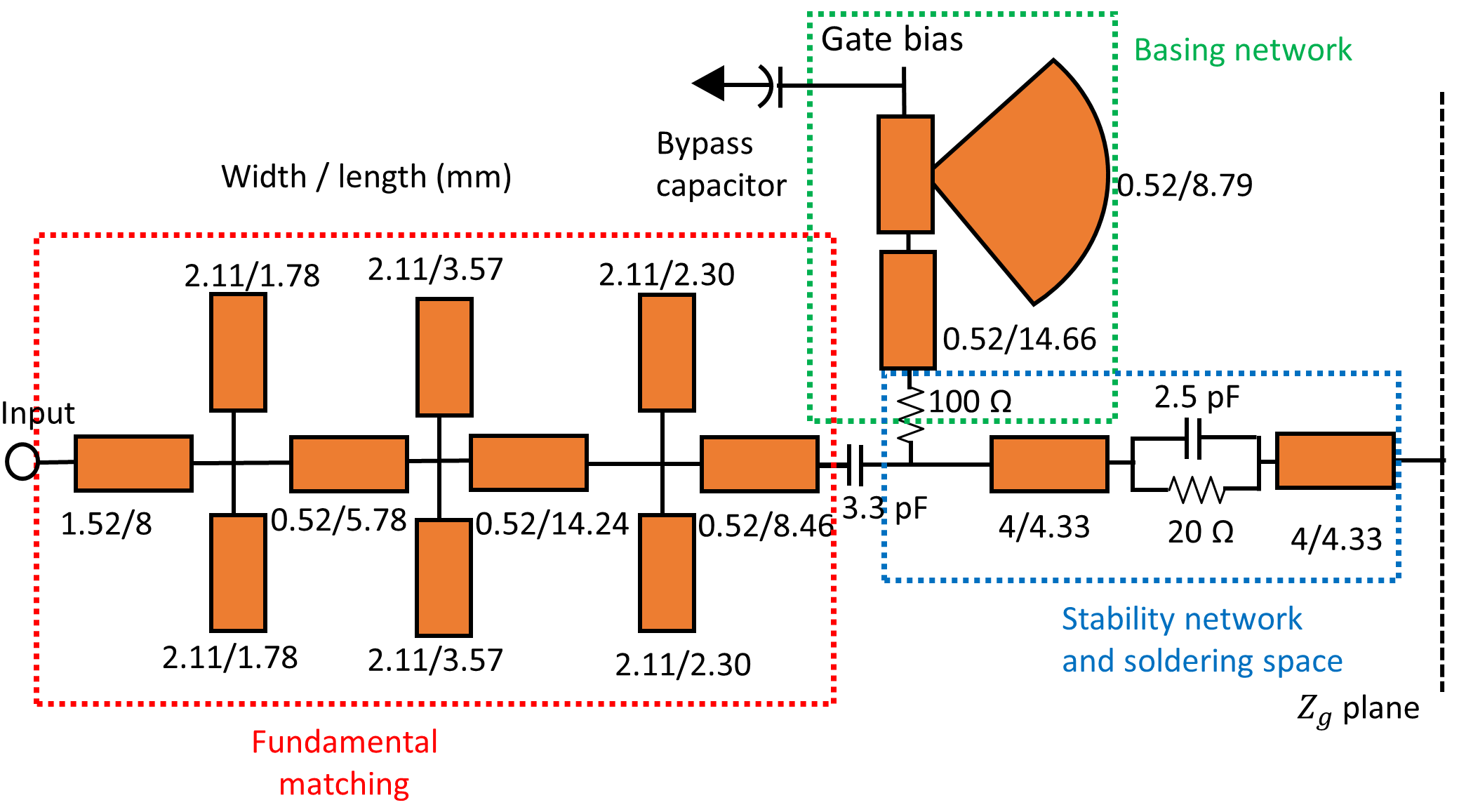

Supplement: S4 Fig — (TIF) [file pone.0306738.s004.tif]

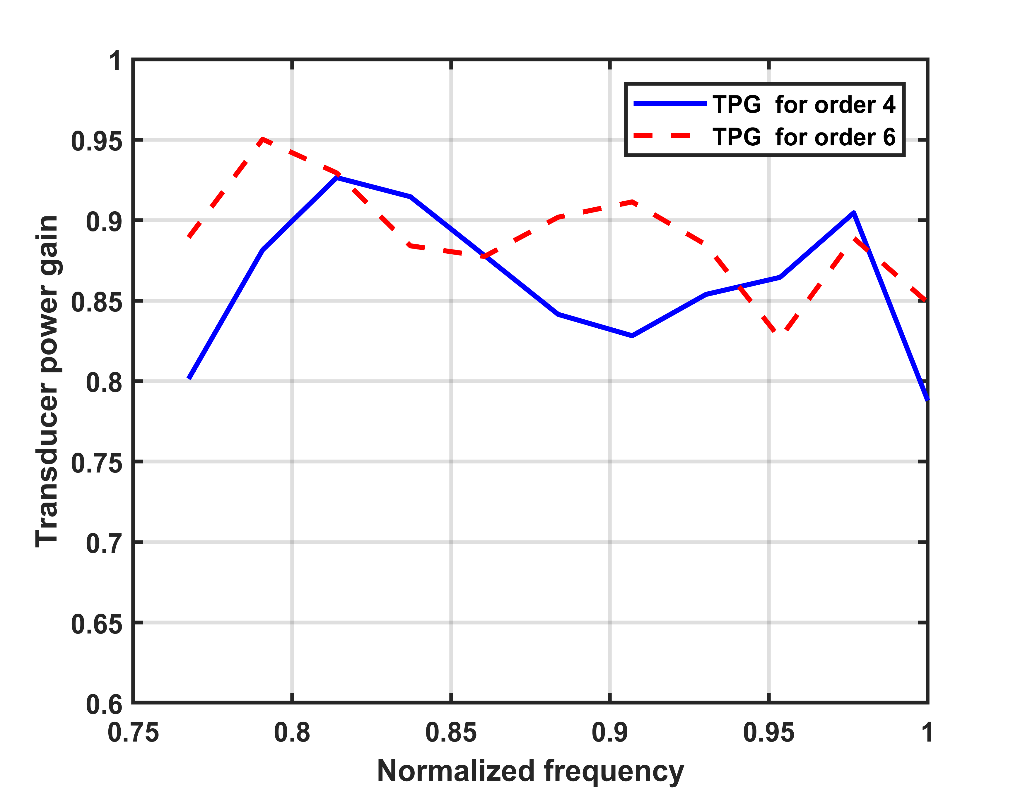

Supplement: S5 Fig — (TIF) [file pone.0306738.s005.tif]

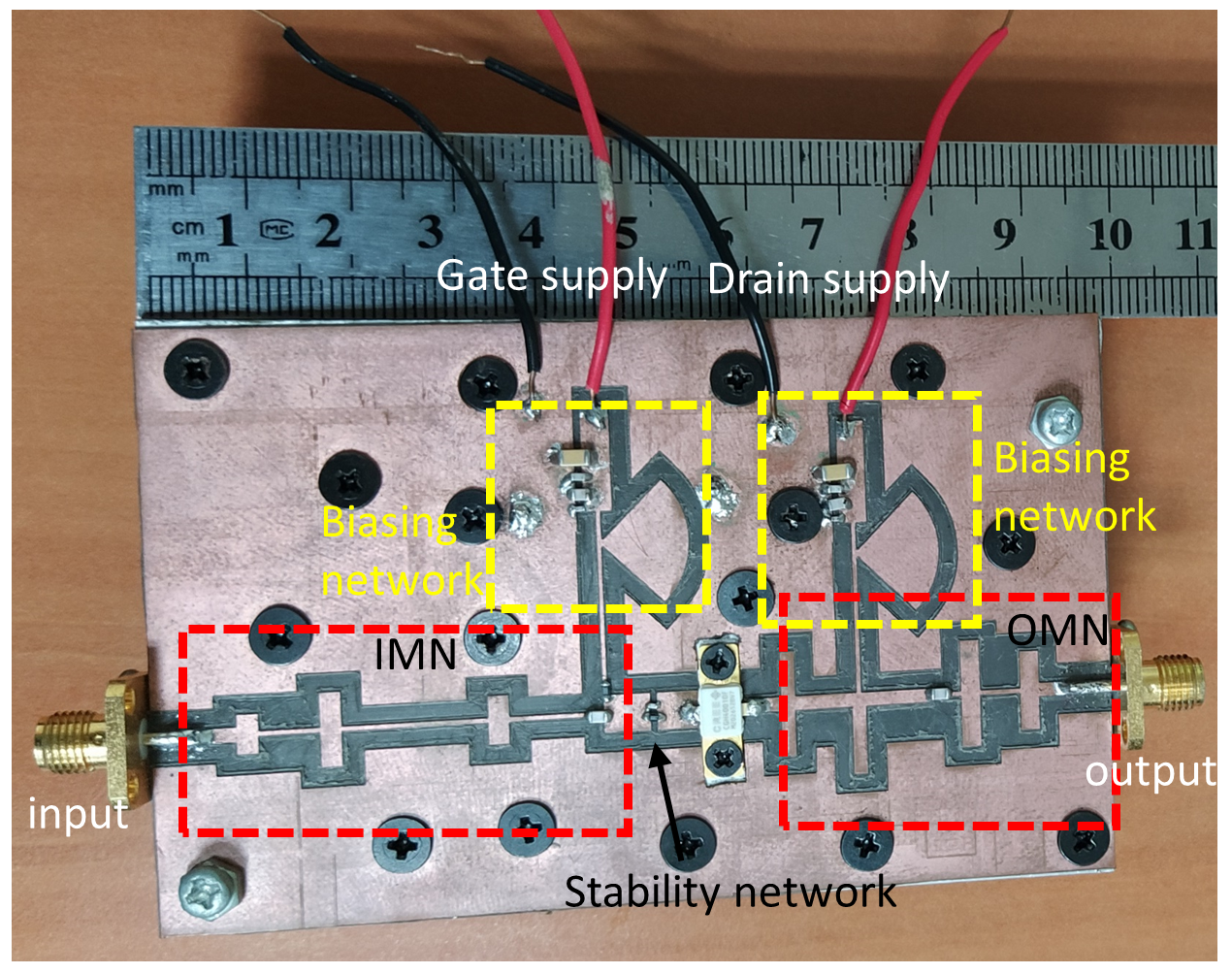

Supplement: S7 Fig — (TIF) [file pone.0306738.s007.tif]

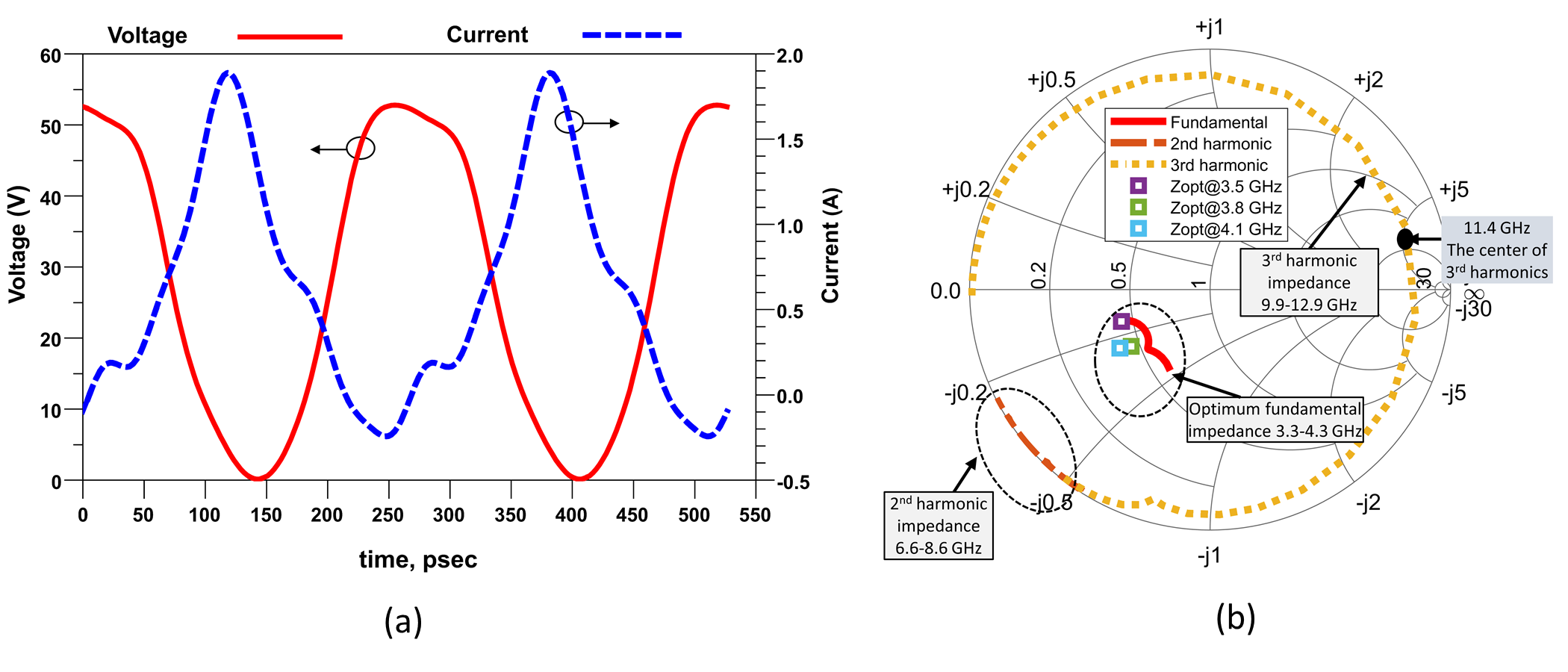

Supplement: S8 Fig — (a) Simulated voltage and current waveform at the I-Gen plane at 3.8 GHz frequency and (b) Impedance trajectories of the OMN at I-Gen plane. (TIF) [file pone.0306738.s008.tif]

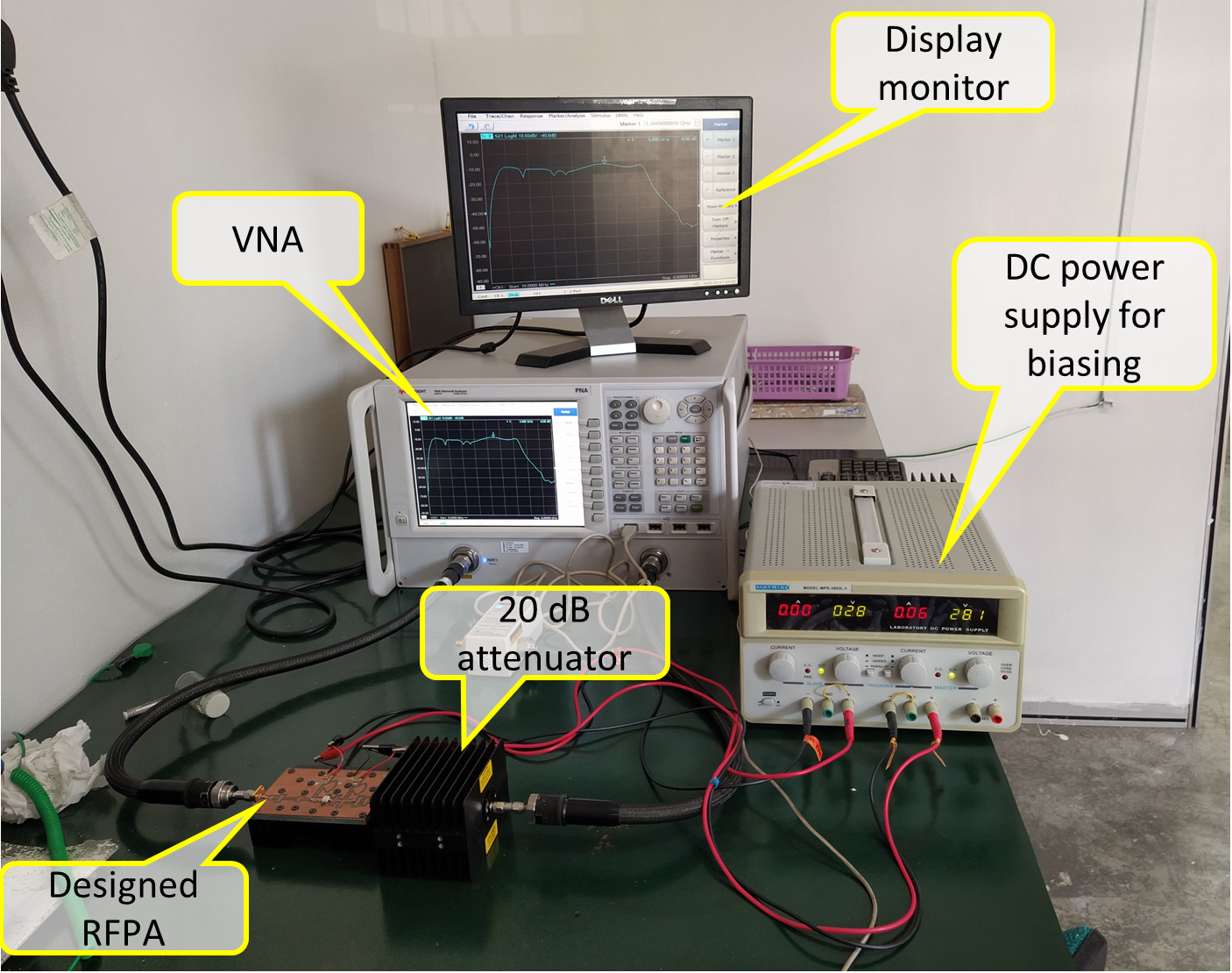

Supplement: S9 Fig — (TIF) [file pone.0306738.s009.tif]

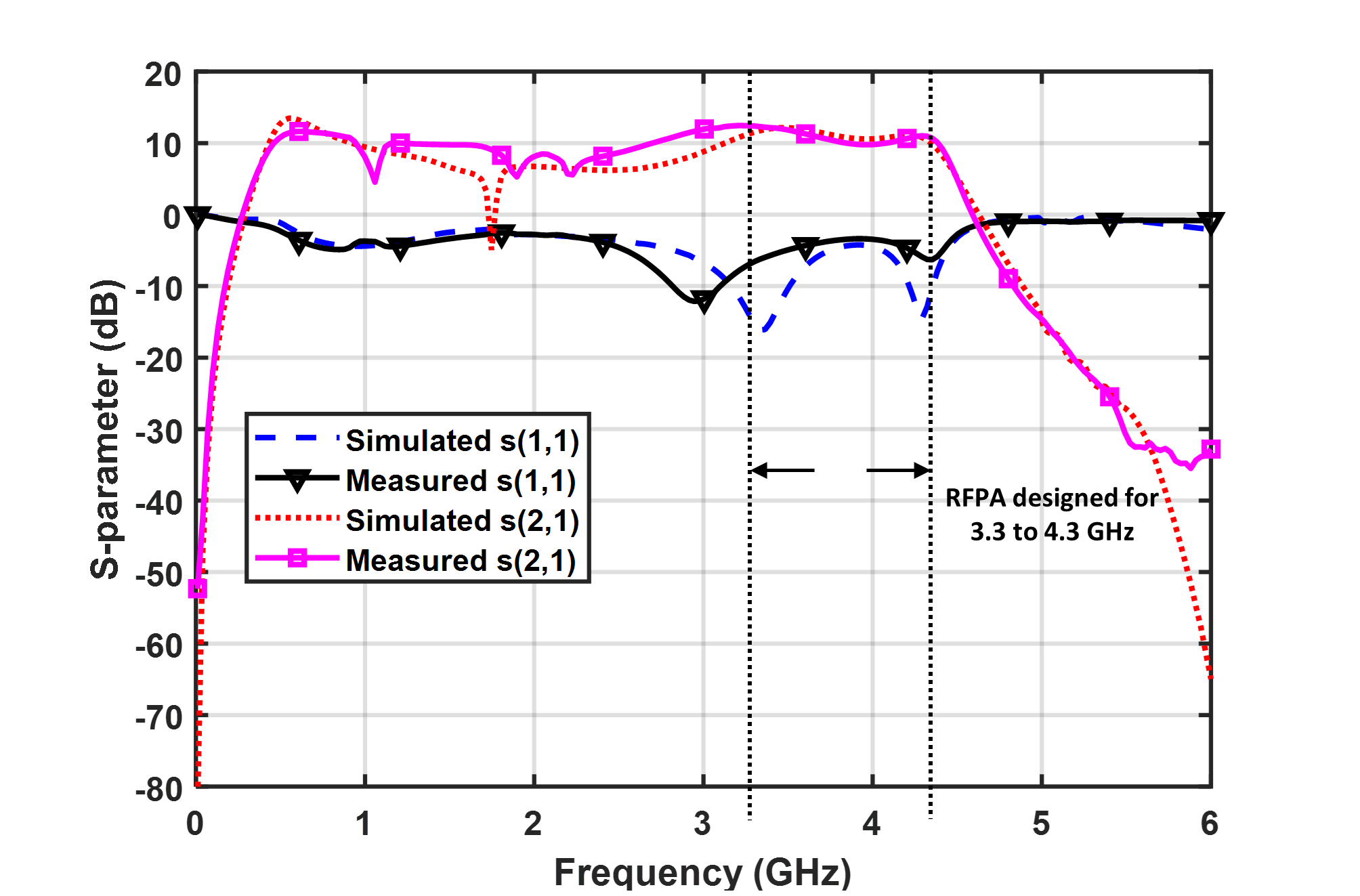

Supplement: S10 Fig — (TIF) [file pone.0306738.s010.tif]

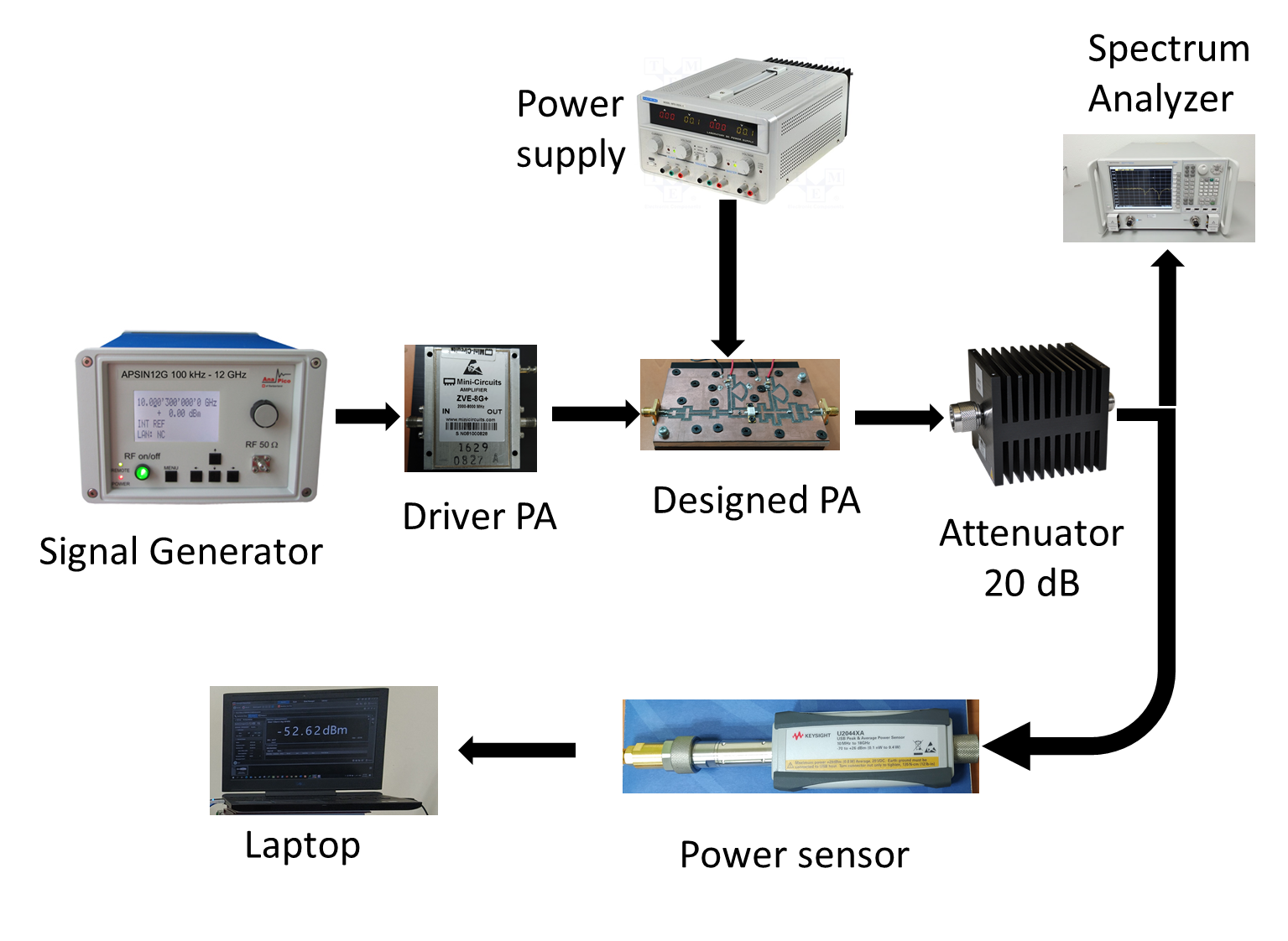

Supplement: S11 Fig — (TIF) [file pone.0306738.s011.tif]

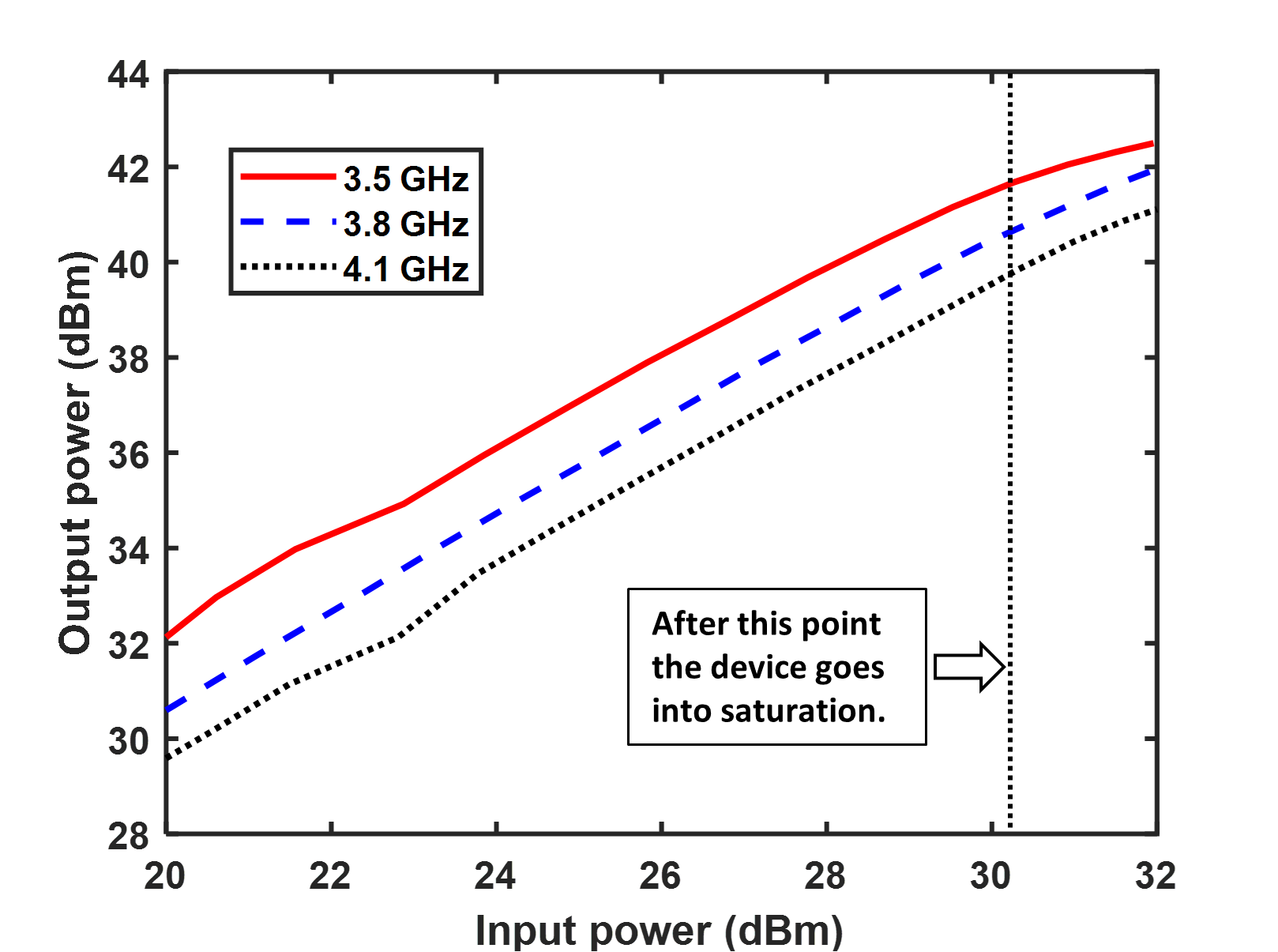

Supplement: S12 Fig — (TIF) [file pone.0306738.s012.tif]

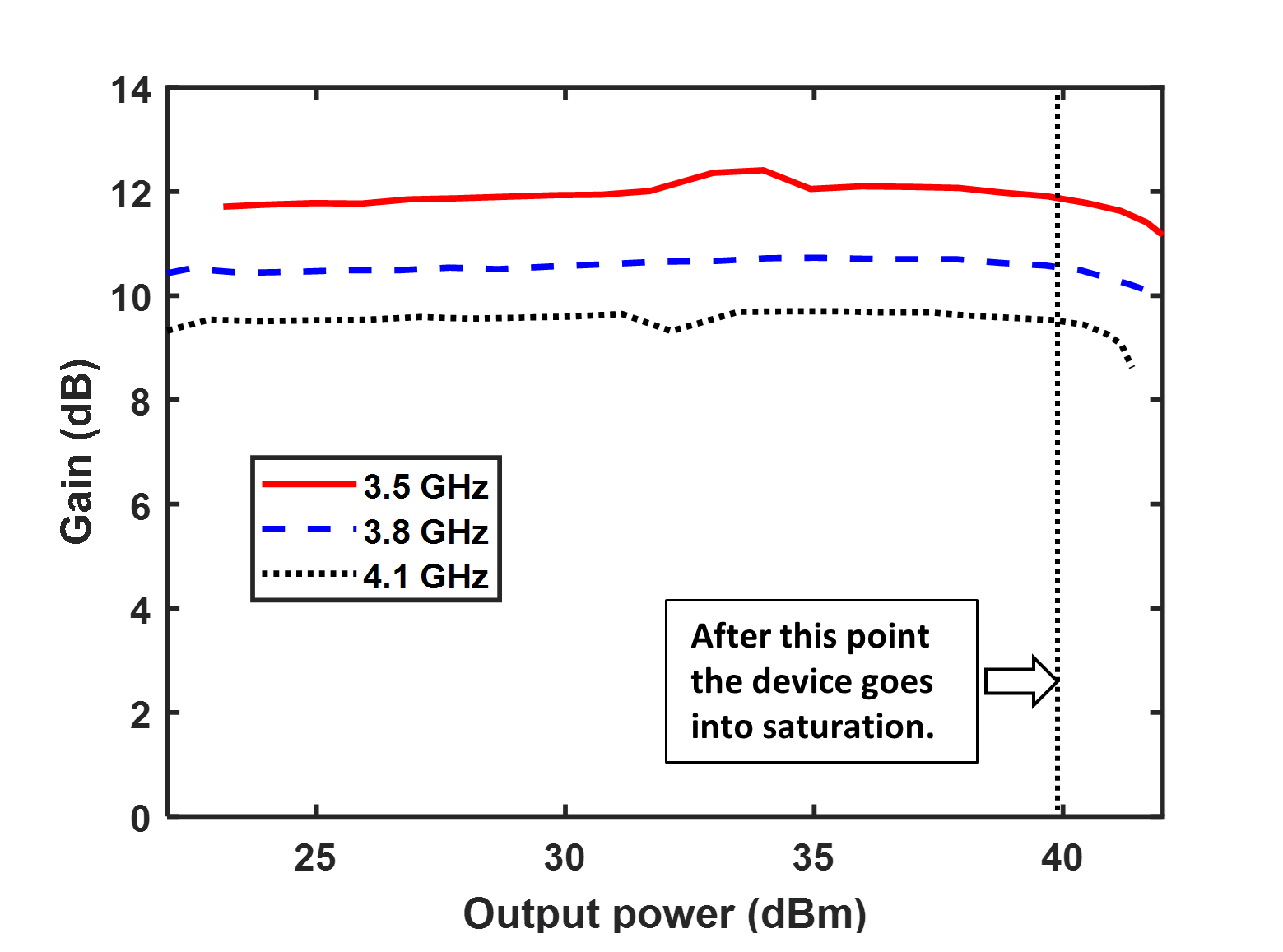

Supplement: S13 Fig — (TIF) [file pone.0306738.s013.tif]

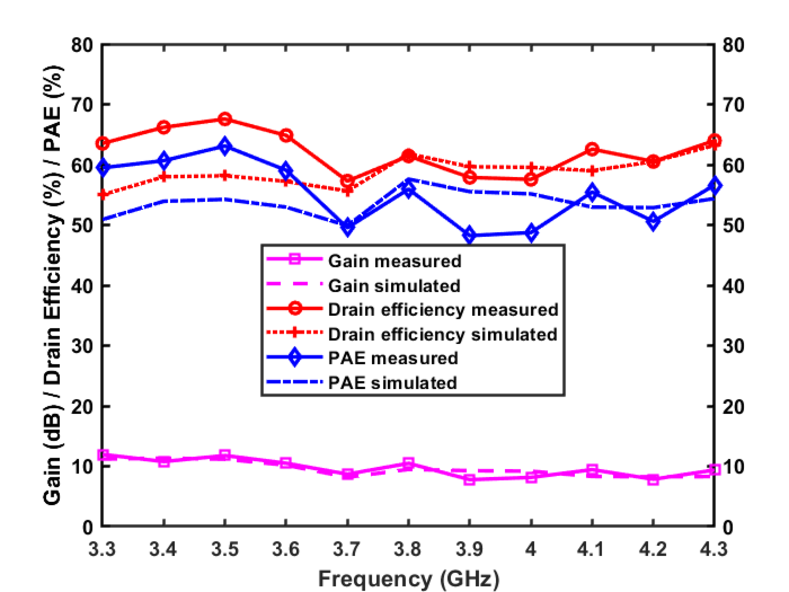

Supplement: S14 Fig — (TIF) [file pone.0306738.s014.tif]

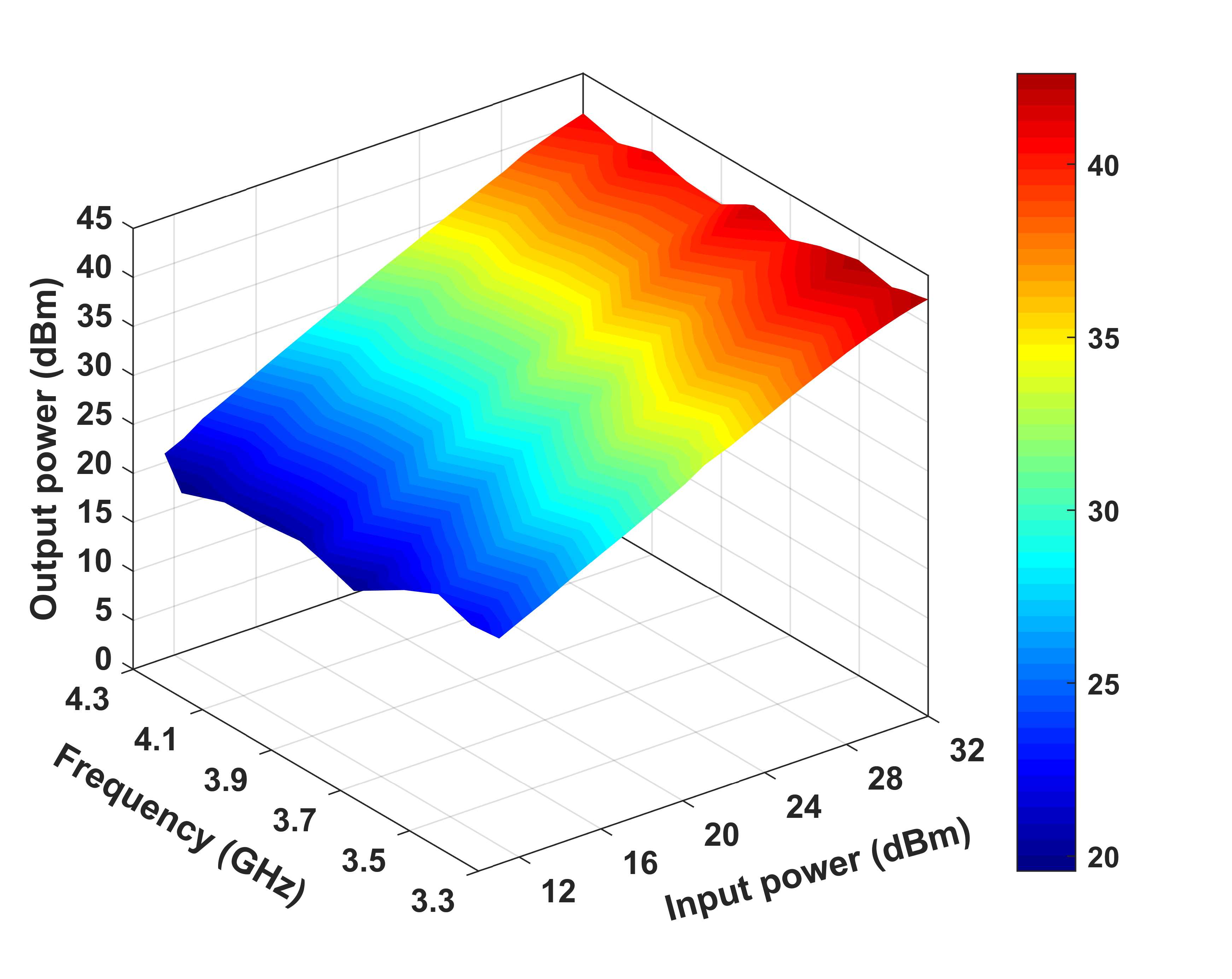

Supplement: S15 Fig — (TIF) [file pone.0306738.s015.tif]

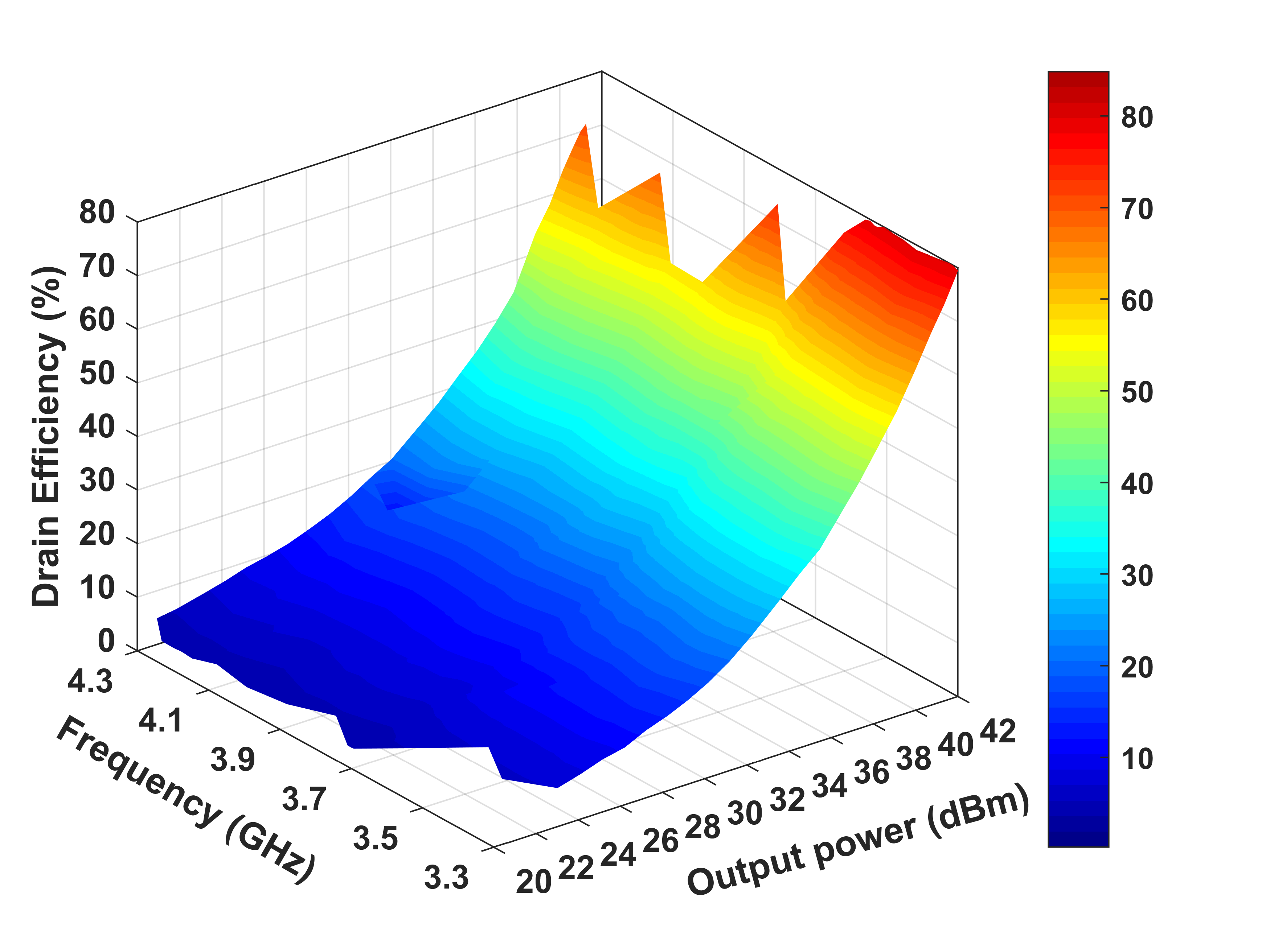

Supplement: S16 Fig — (TIF) [file pone.0306738.s016.tif]

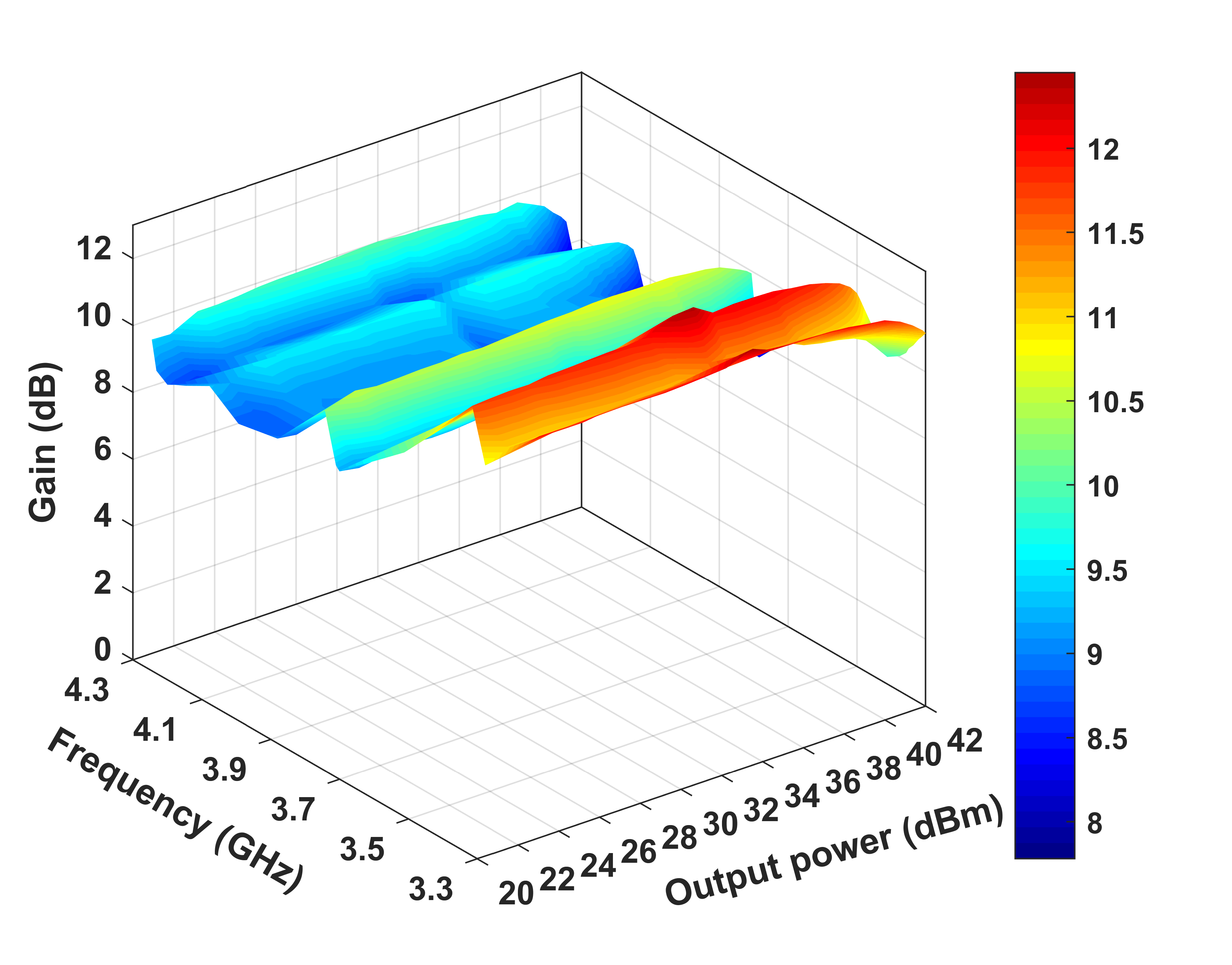

Supplement: S17 Fig — (TIF) [file pone.0306738.s017.tif]

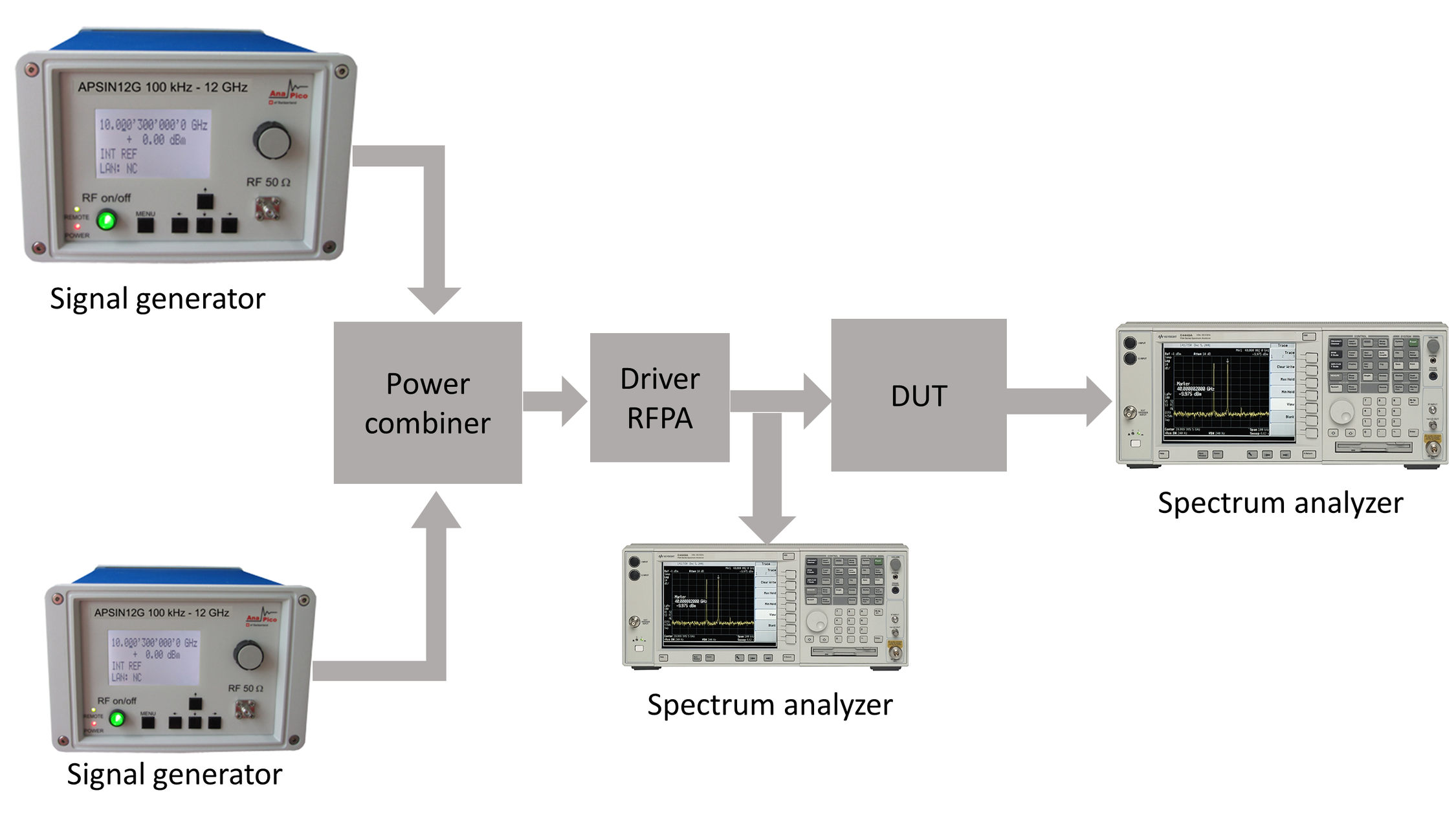

Supplement: S18 Fig — (TIF) [file pone.0306738.s018.tif]

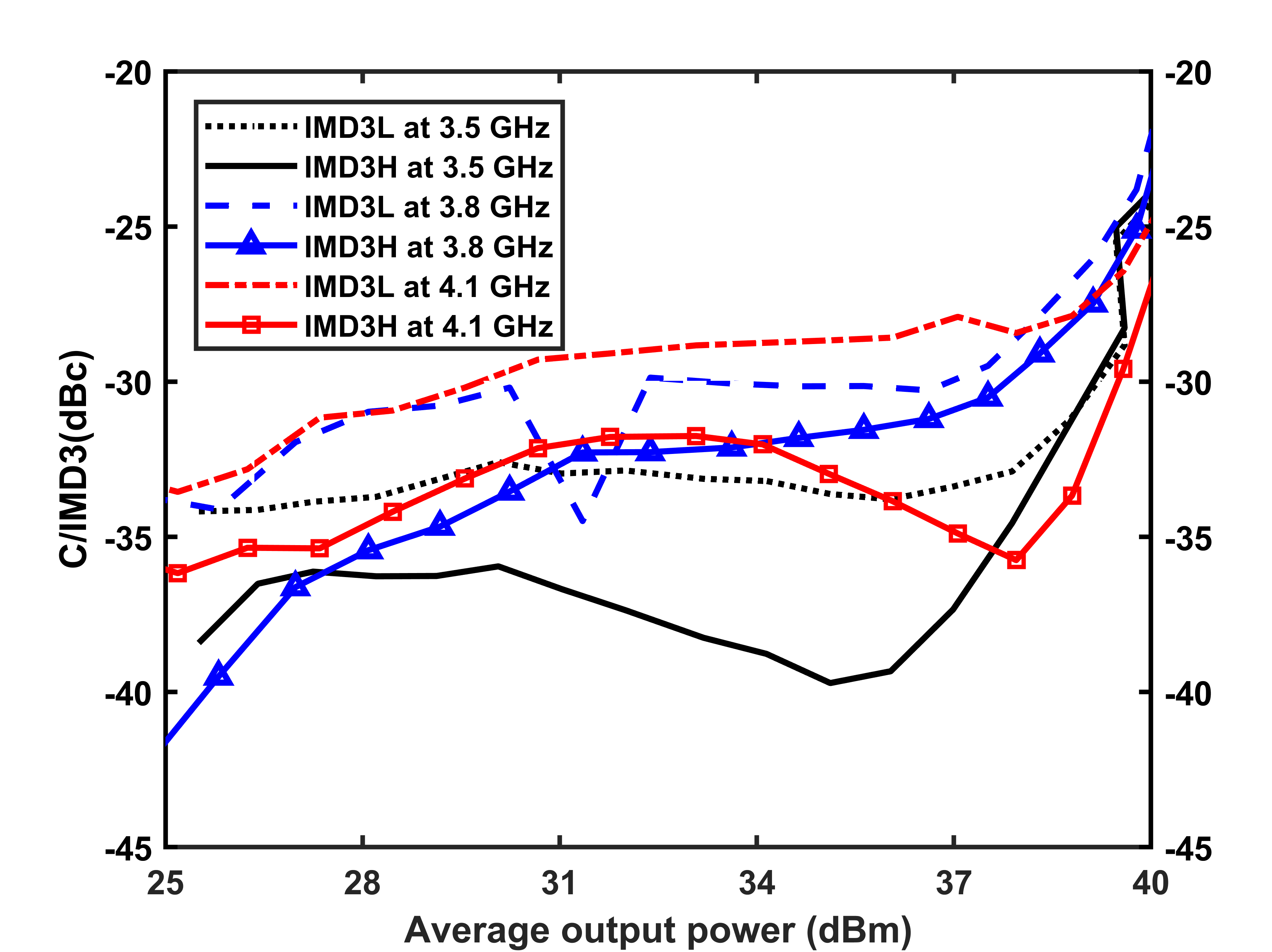

Supplement: S19 Fig — (TIF) [file pone.0306738.s019.tif]

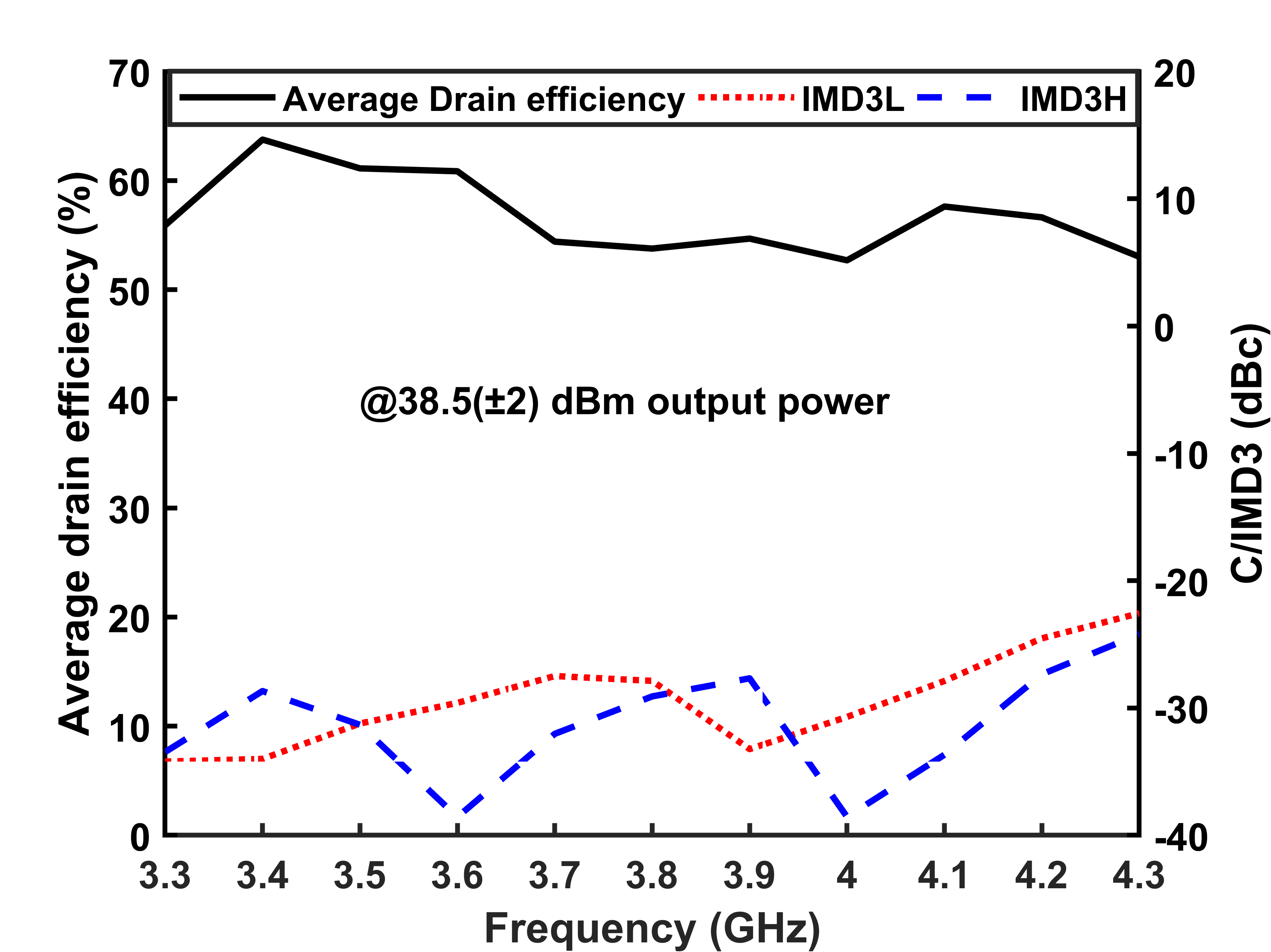

Supplement: S20 Fig — (TIF) [file pone.0306738.s020.tif]
